# Covariation between homeodomain transcription factors and the shape of their DNA binding sites

**DOI:** 10.1093/nar/gkt862

**Published:** 2013-09-27

**Authors:** Iris Dror, Tianyin Zhou, Yael Mandel-Gutfreund, Remo Rohs

**Affiliations:** ^1^Molecular and Computational Biology Program, University of Southern California, Los Angeles, CA 90089, USA and ^2^Department of Biology, Technion – Israel Institute of Technology, Technion City, Haifa 32000, Israel

## Abstract

Protein–DNA recognition is a critical component of gene regulatory processes but the underlying molecular mechanisms are not yet completely understood. Whereas the DNA binding preferences of transcription factors (TFs) are commonly described using nucleotide sequences, the 3D DNA structure is recognized by proteins and is crucial for achieving binding specificity. However, the ability to analyze DNA shape in a high-throughput manner made it only recently feasible to integrate structural information into studies of protein–DNA binding. Here we focused on the homeodomain family of TFs and analyzed the DNA shape of thousands of their DNA binding sites, investigating the covariation between the protein sequence and the sequence and shape of their DNA targets. We found distinct homeodomain regions that were more correlated with either the nucleotide sequence or the DNA shape of their preferred binding sites, demonstrating different readout mechanisms through which homeodomains attain DNA binding specificity. We identified specific homeodomain residues that likely play key roles in DNA recognition via shape readout. Finally, we showed that adding DNA shape information when characterizing binding sites improved the prediction accuracy of homeodomain binding specificities. Taken together, our findings indicate that DNA shape information can generally provide new mechanistic insights into TF binding.

## INTRODUCTION

The recognition of DNA binding sites by transcription factors (TFs) is a critical step in the control of gene expression. We previously suggested that TFs identify their binding sites via two mechanisms: (i) base readout, which involves the recognition of unique chemical signatures at the edges of base pairs, mainly in the major groove (1), and (ii) shape readout, in which the protein recognizes the 3D sequence-dependent DNA shape (2,3). We have shown that the latter plays an important role in the DNA binding of several TF families (4–7) and other DNA binding proteins (8–11). Specifically, we demonstrated that a narrow minor groove width (MGW) can enhance the negative electrostatic potential in the minor groove, which can attract positively charged amino acids such as arginine, lysine (8) and histidine, considering that the latter can be protonated (11).

Over the past decade, a large effort has been made to study the binding preferences of TFs at the high-throughput (HT) level. Studies using experimental HT technologies, such as *in vitro* protein binding microarray (PBM) (12–14), bacterial one-hybrid (B1H) (15,16), HT-SELEX/SELEX-seq (17–20), *in vivo* ChIP-chip (21,22) and ChIP-seq (23), have provided detailed information on TF binding. Whereas many computational approaches have been developed to extract the sequence preferences of TFs from HT binding data, only recently has it become feasible to derive the preferred local structure of TF binding sites in a systematic manner. Although the 3D shape of DNA is dependent on its nucleotide sequence, degeneracy exists between DNA shape and sequence. Different nucleotide composition can give rise to a similar structure, and variations in a single nucleotide can alter the DNA shape in regions of several base pairs. We developed a computational HT approach for predicting the DNA shape features for any given DNA sequence on a genome-wide scale (24). This HT method allows the study of DNA shape recognition by TFs using the increasing number of sequence data sets that have become available from diverse experimental approaches.

In this study, we focused on the homeodomain family of TFs, which is the second most abundant TF family in mammals. Homeodomain proteins have important functions in many aspects of development, such as the patterning of the anterior-posterior axis (25,26) and organogenesis (27) in a large range of organism, including fungi, plants and animals. The 3D structures of homeodomain–DNA complexes (4,28–32) provide insights into the DNA binding mechanisms of homeodomains. Homeodomains consist of ∼60 amino acids that form a three-helix bundle, of which the third helix is the recognition helix. The latter is inserted into the DNA major groove, which provides a unique signature of the functional groups of the bases and accounts for base readout. The flexible N-terminal tail is inserted into the minor groove that, in turn, accounts for shape readout (4,33). We recently showed that the MGW is used to distinguish between the preferred binding sites of the anterior and posterior Hox proteins in *Drosophila* (20).

For decades, a major goal in the field of protein–DNA readout has been the identification of a recognition code, which defines the preferred base pair interactions for key amino acids. In the case of zinc finger proteins, a recognition code was derived and shown to fairly well predict binding specificities (34,35). However, for the homeodomain family, this definition of a recognition code seems to be more complicated. Extensive experimental and computational HT studies have attempted to unravel the role of individual homeodomain residues in determining sequence specificity (14,16,36). A recent study used a reengineered engrailed homeodomain, in which residues of the recognition helix were randomized and selected against a B1H library, resulting in a functionality map of specific positions in the homeodomain (37). Other studies have used information-based approaches to identify pairs of amino acids and nucleotides, demonstrating significant covariation between residues in the recognition helix and nucleotides in the core binding sites (16,38). Overall, although researchers have better defined the roles of individual residues in the recognition helix, the role of the N-terminal tail in determining binding specificity remains poorly understood.

A simple recognition code that connects amino acids with nucleotides might not be applicable for homeodomain–DNA recognition. Nevertheless, studying the sequence-structure degeneracy of DNA might elucidate how binding specificity is achieved for this protein family. Here, we examined the correlations between the amino acid sequences of homeodomains and the corresponding DNA sequence and shape attributes of their binding sites. We have extracted the available sequence binding preferences of homeodomains in mouse and *Drosophila*, from PBM (14) and B1H (16) data, respectively. By comparing the pair-wise similarities of homeodomain sequences with the pair-wise similarities of the sequence and shape of their DNA binding sites, we show that the N-terminal tail plays a general role in achieving binding specificity among the homeodomain proteins via DNA shape recognition. We further conducted statistical analyses to discover specific amino acids in the N-terminal tail of the homeodomain proteins that play a role in shape readout. Finally, we propose a novel homeodomain shape recognition code that defines the preferred DNA shape for key amino acids in the N-terminal tail.

## MATERIALS AND METHODS

### Scoring the amino acid similarity between homeodomains

To quantify the similarities between different homeodomains, we aligned 168 homeodomains from mouse (14) and 84 homeodomains from *Drosophila* (16) using MAFFT (39). We calculated pair-wise similarity scores using BLOSUM45 (40).

### Generation of the position frequency matrix and scoring the nucleotide similarity between DNA binding sites

For the PBM data set, we collected unique 8-mers, each with a relative enrichment score (E-score) ≥0.45 (14). To generate position frequency matrices (PFMs), we used multiple sequence alignment to align all 8-mers to the consensus homeodomain binding motif NNA(Y/K)N, where N can be any nucleotide, Y represents C or T and K represents G or T (Supplementary Figure S1A). Selection between NNAYN and NNAKN was done by comparison with published PFMs (14). In cases where the 8-mers had more than one NNA(Y/K)N motif, we selected the alignment that best fitted the published PFM based on the highest similarity score (14). In all cases, both strands were considered. For the B1H data, we used the published alignment (16) to generate PFMs (Supplementary Figure S1B). We calculated the pair-wise similarity between all pairs of PFMs using the Pearson correlation coefficient (PCC). The PCC varies between −1 and 1, and the maximum score is obtained when two homeodomain PFMs have a perfect similarity. A flowchart representing this analysis is shown in Supplementary Figure S1C.

### Scoring the shape similarity between DNA binding sites

For each position of the aligned binding sites, we predicted four structural features, MGW, propeller twist (ProT), Roll and helix twist (HelT). MGW and ProT were defined for each nucleotide position. Roll and HelT are base pair-step parameters; therefore, they were assigned to dinucleotide positions. For each homeodomain, we defined four structural feature vectors (one for each shape parameter), representing the predicted average shape parameters over all selected 8-mers for each position in the alignment. The DNA shape analysis used our HT method (24) to infer structural features from a library of all 512 unique pentanucleotides, derived from 2121 Monte Carlo simulations (41). We have recently applied this approach to Hox proteins (20), basic helix-loop-helix (bHLH) proteins (7) and the endonuclease DNase I (10).

Next, we calculated a pair-wise similarity score for each pair of homeodomains, using the negative absolute value of the Euclidian distance (–|Euclidian distance|) between structural feature vectors. This step was done separately for each structural feature, resulting in four different DNA shape similarity matrices. The negative absolute value of the Euclidian distance was 0 when two feature vectors matched perfectly and assumed its negative minimum value when the feature vectors differed most. We used the negative absolute value of the Euclidian distance to maintain consistency with the nucleotide similarity scores in which high values represent high similarity. A flowchart representing this analysis is shown in Supplementary Figure S2. Notably, although MGW and ProT are base-pair parameters and Roll and HelT are base pair-step parameters, the comparison of the same parameters between binding sites of different homeodomains was not affected by this definition.

### Statistical analysis

#### Pearson correlation coefficient

**Figure 1.**
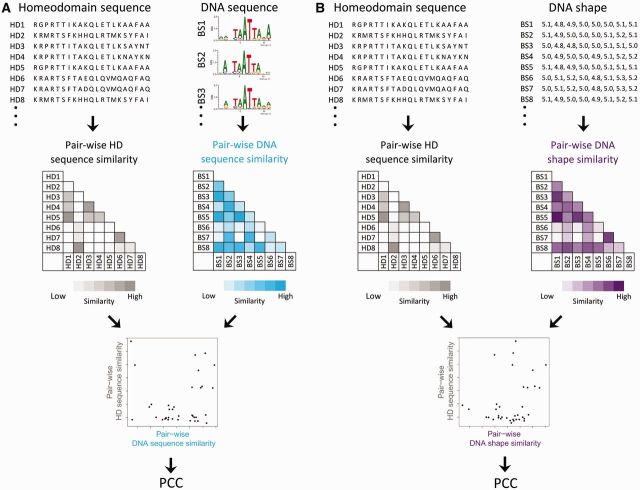
Flowchart for uncovering correlations between the amino acid sequences of homeodomains (HDs) and the nucleotide sequences and structural features of their DNA binding sites (BSs). (A) Based on the sequence alignment of HDs from mouse and *Drosophila*, we calculated two pair-wise similarity scores: one representing the similarity between HD sequences (‘Pair-wise HD sequence similarity’; gray) and another representing the similarity between the nucleotide sequences (shown as PWMs in standardized color code) of their DNA binding sites (‘Pair-wise DNA sequence similarity’; blue). We compared the two similarity scores using PCC. (B) Based on the sequence alignment of HDs from mouse and *Drosophila* and structural features of their DNA binding sites, we calculated five pair-wise similarity scores, one representing the similarity between the HD sequences (‘Pair-wise HD sequence similarity’; gray) and four representing the similarities between DNA shape parameters (MGW, ProT, Roll and HelT) of their DNA binding sites (‘Pair-wise DNA shape similarity’; purple). We compared each set of two similarity scores using PCC.

PCC is a measurement of the linear dependency between two sets of variables. We used the PCC to measure the correlation between the pair-wise homeodomain sequence similarity scores and each of the five pair-wise binding site similarity scores (one pair-wise DNA sequence similarity and four pair-wise DNA shape similarities), with the PCC ranging from −1 to +1. A flowchart representing this analysis is shown in Figure 1. To account for differences in PFM score distribution, we used an additional approach. In the latter, we assigned the DNA PFM scores to seven bins with scores of 0–0.4, 0.4–0.5, 0.5–0.6, 0.6–0.7, 0.7–0.8, 0.8–0.9 and 0.9–1 and selected an equal number of binding sites from each bin (300 and 200 binding sites for the PBM and B1H data set, respectively).

#### Hypergeometric distribution test

We defined hypergeometric scores to examine the presence of basic amino acids as a function of DNA shape. These scores were calculated using the frequency of positively charged residues (arginine, lysine and histidine, which can be protonated) at each amino acid position of the homeodomains (all homeodomain sequences were aligned as described above), and the frequency of occurrence of a narrow MGW at each nucleotide position of the DNA binding sites. We defined the threshold for a narrow MGW as ≤5.12 Å, which was the average MGW of all analyzed 24 879 binding sites in both homeodomain data sets. Notably, this value is below the standard B-DNA value of 5.8 Å because homeodomain binding sites are AT-rich, which increases the tendency of minor groove narrowing (4). The *P*-values were calculated using the cumulative hypergeometric test between each position in the protein and each position in the DNA binding site, using Equation 1:
(1)
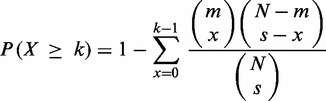



Here, *k* is the number of homeodomains that contain positively charged amino acids and whose binding sites exhibit a narrow MGW; *s* is the number of homeodomains that contain positively charged amino acids at the corresponding position; *m* is the number of homeodomains that exhibit a narrow MGW at that specific position of the binding sites; and *N* is the total number of homeodomains.

#### Mutual information

We defined a mutual information (MI) score between each amino acid position in the protein and each nucleotide position in the DNA binding site, comparing the amino acids with MGW. To compute the MI score, we partitioned the continuous structural features into a discrete alphabet, using a threshold to distinguish a ‘narrow’ from a ‘not narrow’ MGW. The threshold between ‘narrow’ and ‘not narrow’ was defined as the average MGW over all positions in the homeodomain binding sites of the combined mouse and *Drosophila* data sets (5.12 Å). The MI score between each amino acid position in the protein and each nucleotide position in the binding site was calculated using Equation 2:
(2)
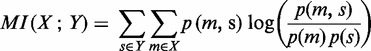



Here, *p(m,s)* is the joint probability for the occurrences of *m* and *s*; and *p(m)* and *p(s)* are the probabilities of the independent occurrences of *m* and *s* in *X* and *Y*, respectively.

### Multiple linear regression and receiver operator characteristic curves

We used multiple linear regression (MLR) with 10-fold cross validation to predict all 8-mer E-scores for each homeodomain in the mouse data set (14). E-score is a measurement for the relative binding affinity of a protein to a certain 8-mer derived from universal PBM experiments (42). To predict E-scores, the 8-mers were randomly assigned to 10 groups, with 9 groups representing the training data set. Using the training data set, we fitted a model to predict the E-scores of the remaining group of 8-mers (test data set). This procedure was repeated 10 times, each time using one of the 10 groups as the test data set, until the E-score of each 8-mer was predicted. The E-score prediction was repeated 50 times, to account for biases from selecting the training and test data sets.

We trained three different MLR models: one model incorporating the nucleotide sequence of each 8-mer as the input, a second model incorporating the sequence and the four shape parameters of each 8-mer as input variables and a third model using the sequence and randomly shuffled shape parameters. As the response variable, we used the E-score of each 8-mer. The 8-mer alignment can result in missing information in both flanks of the binding site. When this occurred, we extended the sequences using a randomized nucleotide selection procedure, assigning an average value for the corresponding shape parameter to the added position.

To measure the predictive power of each model, we calculated the average *R*^2^ (using the squared PCC defined above) between the predicted and experimental E-scores over 50 iterations. We used receiver operator characteristic (ROC) curves to assess the accuracy of the MLR-based prediction in detecting bound and unbound 8-mers. In agreement with (14), we defined the set of bound sequences as all 8-mers with experimental E-scores ≥0.45 (true positives) and the set of unbound sequences as all 8-mers with experimental E-scores <0.45 (true negatives).

## RESULTS AND DISCUSSION

### Correlation between homeodomains and their DNA binding sites

To study the relationship between homeodomains and their preferred binding sites, we concentrated on HT binding data from two independent experiments. The first set of preferred 8-mer sequences of 168 homeodomains in mouse was extracted from PBM data (14). The second set of preferred sequences for 84 homeodomains in *Drosophila* was generated in an independent study using the B1H system (16).

**Figure 2.**
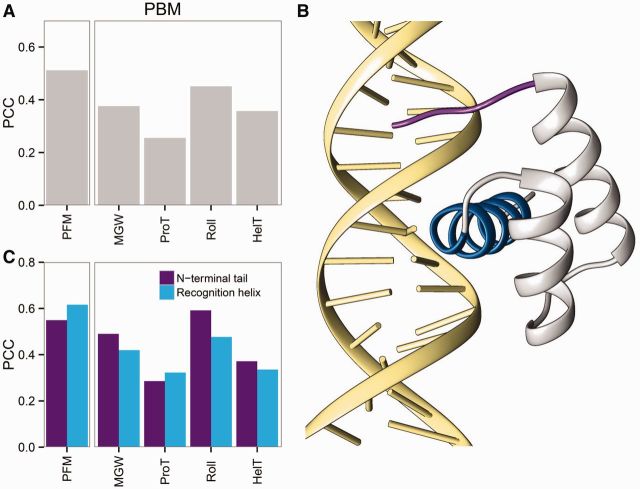
Correlation between homeodomain sequences and the DNA sequences and shapes of their binding sites. (A) PCC between pair-wise HD sequence similarity and pair-wise DNA sequence and shape similarity are shown for HDs in mouse derived from PBM data (14). (B) Co-crystal structure of engrailed HD in complex with DNA (PDB ID 3HDD) (29). Purple represents the N-terminal tail, and blue highlights the recognition helix. (C) PCCs for the comparison of pair-wise HD sequence similarity scores, using only the sequence of the N-terminal tail (purple) or the recognition helix (blue), with pair-wise DNA sequence and shape similarity scores. PBM data for 168 mouse HDs (14) were used in this analysis.

We calculated the pair-wise similarity scores between all pairs of homeodomains (‘Pair-wise HD sequence similarity’), based on their amino acid sequences (Figure 1A) using the BLOSUM45 matrix (see ‘Materials and Methods’ section). We calculated the pair-wise similarity scores between the DNA sequences of the homeodomain binding sites (‘Pair-wise DNA sequence similarity’) by comparing the preferred binding sequence of each homeodomain represented as a PFM. We derived the latter by measuring the PCC between each pair of PFMs (Figure 1A). We calculated the PCC between the homeodomain sequence similarity and the DNA sequence similarity. We found a significant correlation between the two similarity scores (Figure 2A; Supplementary Figure S3A), with PCC values of 0.51 (*P* < 10^−^^16^) for the PBM data set and 0.64 (*P* < 10^−^^16^) for the B1H data set. Our results are comparable with previously reported correlations (PCC of 0.416) between amino acid sequences of 100 homeodomains from different organisms and the nucleotide sequences of their corresponding binding sites (43).

Next, we asked whether the shapes of the DNA binding sites alone correlated with the amino acid sequences of the homeodomains. Among the DNA shape parameters, we focused on MGW, as we previously observed that MGW plays a role in achieving homeodomain binding specificity (4,8,20). We included other relevant shape parameters (ProT, Roll and HelT) (44) that reportedly affect DNA shape readout (7,10,41). To characterize the four DNA shape parameters at each position in the preferred binding sites of each homeodomain, we used our HT prediction method (24). For each homeodomain, we assigned four structural feature vectors (one for each shape parameter), representing the predicted shape parameter as a function of nucleotide sequence. Consequently, we calculated four sets of pair-wise similarity scores by measuring the Euclidian distances between structural feature vectors of all pairs of homeodomains termed ‘Pair-wise DNA shape similarity’ (Figure 1B).

We compared the homeodomain similarity scores with the four DNA shape similarity scores using PCC. Again, we noticed a high correlation between the pair-wise similarity of the homeodomain sequences and the pair-wise DNA shape similarity of their corresponding binding sites for three of the shape parameters (MGW, Roll and HelT) in the PBM data set (PCC of 0.37, 0.45 and 0.35, respectively, all *P* < 10^−^^16^) and B1H (PCC of 0.53, 0.43 and 0.40, respectively, all *P* < 10^−^^16^) (Figure 2A and Supplementary Figures S3 and S4). For ProT, we found a correlation only in the B1H data set (PCC of 0.35, *P* < 10^−^^16^). To account for differences in the occurrence of the similarity scores, we further binned the PFM scores and selected an equal number of sequences from each bin. Consistent with the previous results we did not observe any significant changes in the correlations (Supplementary Figure S5). To assess the significance of these results, we randomly shuffled the homeodomain sequences 1000 times, each time creating a new homeodomain sequence similarity. We measured the PCC between each of the shuffled homeodomain sequence similarities with the DNA sequence and four DNA shape similarities. As expected, we found no significant correlation in the randomly shuffled data, with an average PCC ranging from −0.001 to 0.001 for all five comparisons.

Based on experimental structures of several homeodomain proteins in complex with their DNA targets (4, 28–30, 45), the recognition helix of the homeodomains is thought to be engaged in base readout of the major groove, whereas the N-terminal tail is involved in shape readout of the minor groove (2,4,33) (Figure 2B). Thus, we anticipate high correlations between the protein residues in the recognition helix and the sequence of the DNA binding sites and between the protein residues at the N-terminal tail and the DNA shape parameters. To explore these assumptions, we recalculated the pair-wise homeodomain sequence similarity scores for each of these protein regions independently: namely, considering either the amino acids in the recognition helix only, or the amino acids in the N-terminal tail alone. To define the recognition helix and the N-terminal tail, we used the crystal structure of the engrailed homeodomain (PDB ID 3HDD) (Figure 2B), wherein positions 47–55 denote the recognition helix and positions 1–9 define the N-terminal tail (29).

When we considered each of the two regional homeodomain sequence similarity scores separately, we found that the correlation for DNA sequence was higher with the homeodomain sequence similarity derived from the recognition helix alone, whereas the MGW and Roll similarities were better correlated with the homeodomain sequence similarity derived from only the N-terminal tail (Figure 2C and Supplementary Figure S3A). Similar results were obtained when using position weight matrices (PWMs) instead of PFMs to calculate the PCC (Supplementary Figure S3B). These findings support our previous studies suggesting that the N-terminal tail of homeodomains is involved in DNA shape readout (4,20) and reinforce the idea that DNA shape recognition via the N-terminal tail may generally contribute to the DNA binding specificity of the homeodomain family of TFs.

### Inferring dependencies between homeodomain residues and DNA shape

**Figure 3.**
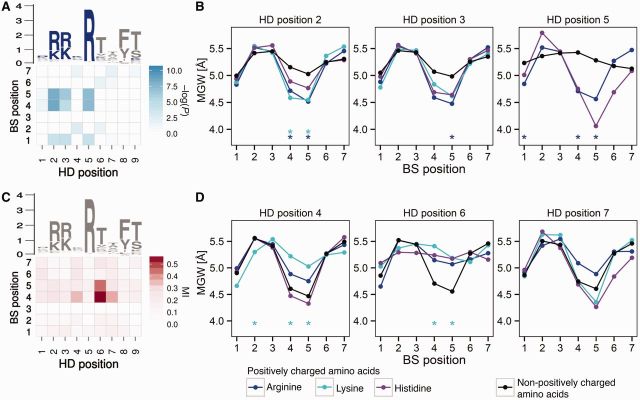
Dependencies between N-terminal tail residues (amino acid positions 1–9) and MGW of the DNA binding sites based on PBM data. (A) A heat map (blue) shows −log of the *P*-values of hypergeometric scores between positively charged amino acids (highlighted in blue) and narrow minor groove regions at each position of the HD-DNA binding sites derived from PBM data for 168 mouse HDs (14). Sequence logos representing the alignment of these 168 HD sequences are shown above. Basic amino acids in the logos are highlighted in blue. Numbering of the amino acid positions corresponds to the convention for Hoxa9 in mouse (32). (B) For the three HD positions with the highest hypergeometric scores (residues 2, 3 and 5), the average MGW for the DNA binding sites of all HDs with arginine (blue), lysine (cyan), histidine (purple) and non-positively charged amino acids (black) is plotted. Asterisks (color code referring to amino acid type) indicate nucleotide positions with significant differences in MGW (Wilcoxon *P* < 5 × 10^−5^). (C) A heat map (maroon) shows MI scores between each amino acid position in the HDs and MGW at each nucleotide position of their preferred DNA binding sites for the PBM data from mouse (14). Sequence logos representing the alignment of all 168 homeodomain sequences in mouse are shown above. Numbering of the amino acids corresponds with the convention for Hoxa9 in mouse. (D) For the three positions with the highest MI scores (residues 4, 6 and 7), the average MGW for the binding sites of all HDs with arginine (blue), lysine (cyan), histidine (purple) and non-positively charged amino acids (black) is plotted. Asterisks (color code referring to amino acid type) indicate nucleotide positions with significant differences in MGW (Wilcoxon *P* < 5 × 10^−5^).

We previously demonstrated that the mechanism by which DNA-binding proteins recognize DNA shape involves electrostatic attraction between positively charged residues and the shape-dependent negative electrostatic potential of the minor groove (8). Therefore, we sought to identify the homeodomain residues that are involved in this kind of DNA shape readout mode, by searching for coappearance of basic amino acids with narrow minor groove regions at given nucleotide positions. Using the PBM data set and, to a lesser extent, the B1H data set, we found a significantly high correlation between the positively charged residues at amino acid positions 2, 3 and 5 of the N-terminal tail with a narrow minor groove at nucleotide positions 1, 4 and 5 of the binding sites (Figure 3A, Supplementary Figure S6 and Supplementary Tables S1–S2). Interestingly, all three residues were previously shown to be involved in DNA shape recognition based on the analysis of independent homeodomain–DNA complexes (4,8). We obtained consistent results when we used different thresholds to define a narrow MGW (4.9, 5.0, 5.1 and 5.2 Å).

Next, we separately analyzed individual residues in the N-terminal tail to determine if different amino acids in these positions would lead to different DNA shape preferences of the homeodomains. We clustered the homeodomains based on their amino acid similarity at positions 2, 3 or 5 of the N-terminal tail and calculated the MGW preferences for each cluster (Figure 3B). Homeodomains with an arginine at position 5 seemed to significantly prefer binding sites with a narrower MGW at nucleotide positions 1, 4 and 5, compared with homeodomains with a non-positively charged amino acid at position 5 (Wilcoxon *P* < 5 × 10^−^^5^). Furthermore, homeodomains with an arginine or lysine at position 2 or 3 seemed to prefer targets with a narrower MGW at nucleotide position 5, compared with homeodomains with no positively charged amino acid at position 2 or 3.

A previous study stressed the importance of an alanine at position 8 by showing that mutating alanine to phenylalanine in that position seemed to change the binding specificity of Caup homeodomains (16). Although the alanine at position 8 does not penetrate into the minor groove, the previous study suggested that this mutation influences the conformation of the N-terminal tail over the minor groove and, thus, changes the DNA binding specificity without directly interacting with the minor groove (16). To identify the residues that are important for DNA shape readout but that do not necessarily recognize the minor groove directly, we calculated the MI score between the protein residues and the DNA shape of their binding site. Previous studies have used MI to study the correlation between amino acids in the homeodomain and the nucleotide sequence of their binding sites (16,36,38). Using HT DNA shape prediction, we were able to gain further insights into the relationship between the homeodomain sequence and the shape of their DNA binding sites.

We partitioned the continuous shape values into discrete bins by using the average MGW over all sequences and positions (5.12 Å) as the threshold for a narrow MGW. Then, we calculated the MI score between occurrences of amino acids at each position in the homeodomains and each nucleotide in the DNA binding sites, which were assigned a narrow or wide MGW (Figure 3C). We ranked the correlations between each amino acid position and each nucleotide position based on their MI scores, with a high MI score suggesting covariance. The resulting MI score matrix indicated that specific homeodomain residues correlated with preferred structural features of their binding sites (Figure 3C for N-terminal tail residues; Supplementary Figure S7 for all residues; Supplementary Tables S3–S4). Specifically, we found the highest MI scores between residues 4, 6 and 7 in the N-terminal tail of the homeodomains and the MGW at positions 4 and 5 in the binding sites in the PBM and B1H data sets, reemphasizing the importance of these amino acid positions for DNA shape readout. To verify that these results were not dependent on the threshold used for a narrow MGW, we repeated the analysis using different thresholds ranging from 4.9 to 5.2 Å. We found high MI scores between residues 4, 6 and 7 when lowering the threshold to 4.9, 5.0 and 5.1 Å. Raising the threshold to 5.2 Å resulted in only one high MI score at position 6. We observed high MI scores for positions in the recognition helix that are known to be important for base readout (e.g., positions 50 and 54). This finding likely reflects the dependency of structural features on the DNA sequence.

Zooming into the residues with the highest MI scores in the N-terminal tail (residues 4, 6 and 7) (Figure 3D), we noticed that homeodomains with positively charged amino acids at position 6 seemed to prefer binding sites with wider MGW at nucleotide positions 4 and 5, compared with homeodomains without a basic amino acid at position 6 (Wilcoxon *P* < 5 × 10^−^^5^ for homeodomains with lysine at position 6). The preference for a positively charged amino acid at position 6 to bind to sequences with a wider MGW, rather than a narrow MGW, indicates that these positions are not directly involved in interactions with the DNA but instead assist in positioning the N-terminal tail. Furthermore, we noticed that homeodomains with a lysine at position 4 seemed to significantly prefer binding sites with a narrower MGW at nucleotide position 2 and wider MGW at positions 4 and 5 of their DNA binding sites. However, we could not find any distinct shape preference for a positively charged amino acid at position 7.

### Deriving a shape recognition code for homeodomains

**Figure 4.**
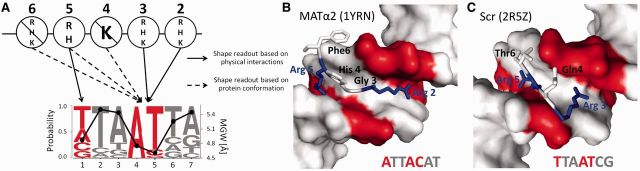
Homeodomain–DNA shape recognition code. (A) We defined a DNA shape recognition code based on interactions between amino acids in the N-terminal tail (top) (HD positions 2–6) and the preferred MGW at each position of the DNA binding site (bottom). The average MGW of all homeodomains in mouse is plotted along the logo representing the combined DNA sequence and shape preference of all homeodomains [using the mouse PBM data (14)]. Nucleotide pairs (red) represent regions of the narrow minor groove. A diagonal line over the amino acid labels at positions 4 and 6 indicates amino acids that are correlated with a wider minor groove. (B) Co-crystal structure of MATα2 in complex with MATa1 and DNA (PDB ID 1YRN) (30). Shown here are residues 2–6 of the N-terminal tail with amino acids at positions 2 and 5 (blue) engaged in physical interactions with DNA. Nucleotide pairs 1, 4 and 5 (red) represent regions with a narrow minor groove. (C) Co-crystal structure of Sex combs reduced (Scr) in complex with Exd and DNA (PDB ID 2R5Z) (4). Shown here are N-terminal tail residues at positions 3–6 with amino acids 3 and 5 (blue) engaged in physical interactions with DNA. Nucleotide pairs 1, 4 and 5 (red) represent regions with a narrow minor groove.

The aforementioned findings suggest a set of recognition rules for the key amino acids in the N-terminal tail (Figure 4A). Overall, we identified a clear preference of the homeodomain family to select sites possessing two narrow minor groove regions at nucleotide positions 1 and 4–5. The selection of minor groove geometry, which is a general feature of the entire family, is most likely due to basic residues in the N-terminal tail, whereas differences in shape preferences within the homeodomain family are due to amino acid variations in these N-terminal residues. We can further divide the amino acid positions in the N-terminal tail into two groups. The first group includes positions wherein basic residues correlate with a narrow MGW and are engaged in DNA shape readout through physical interactions. In contrast, the second group includes positions wherein residues are not necessarily engaged in direct physical interactions with the DNA. However, these residues correlate with DNA shape preferences, possibly influencing the protein conformation at the interface.

#### Shape readout based on physical interactions

We previously showed that a narrow MGW enhances the negative electrostatic potential within the minor groove, which assists in attracting positively charged amino acids (8). In this study, using hypergeometric tests, we detected high correlations between narrow minor groove regions in the binding site and the presence of basic amino acids at the N-terminal positions 2, 3 and 5. This result suggests that the residues at these positions play key roles in shape readout through interactions with the shape-dependent properties of the DNA. Positions 2, 3 and 5 in the N-terminal tail are known to be important for homeodomain–DNA recognition. All three positions were shown to be inserted into narrow minor groove regions in co-crystal structures of various homeodomain–DNA complexes: namely, MATa1-MATα2 (30) (Figure 4B), Exd-Scr (4) (Figure 4C), Exd-Ubx (28) and Oct-1 (46).

Homeodomains with positively charged amino acids at position 2 and 3 seemed to prefer sequences with narrow minor groove regions at nucleotide positions 4–5, compared with homeodomains that do not possess basic amino acids at these positions. These residues have been shown to be inserted into the minor groove in several co-crystal structures (Figure 4B and C) (4,29,30,32). Homeodomains with basic amino acids at position 5 seemed to prefer sequences with two narrow minor groove regions at nucleotide positions 1 and 4–5. In contrast, homeodomains without a basic amino acid at this position seemed to lack a strong minor groove geometry preference. The high conservation of arginine at position 5 reinforces the importance of this position at the N-terminal tail. Arginine 5 has been shown to insert into the minor groove at the 5′ end of the binding site in many homeodomain–DNA complexes (Figure 4B and C) (4, 29–32). Whereas previous studies have observed the electrostatic interactions between the amino acid at position 5 of the N-terminal tail and nucleotide position 1 in the binding site (4,20), we also found interactions of this amino acid position with nucleotide positions 4 and 5 in the binding site. These interactions likely are mediated through conformational properties of the protein, or are a result of a dependency between amino acid position 5 and other amino acid positions.

#### Shape readout based on protein conformation

Using MI, we identified high correlations between amino acids at positions 4, 6 and 7 of the N-terminal tail and the MGW of their binding sites. The fact that we did not observe a high correlation between narrow MGW and basic amino acids at these positions suggests that these residues may be involved in DNA recognition without direct physical interactions. We hypothesize that these residues are involved in placing the flexible N-terminal tail in the correct orientation that allows for the insertion of key amino acids at positions 2, 3 and 5 into the minor groove.

Homeodomains with a lysine at position 4 seemed to prefer binding sites with a wider MGW at nucleotide positions 4–5. This observation is consistent with our recent HT shape analysis of *Drosophila* Hox protein binding sites (20). In the latter study, we found that anterior Hox proteins in complex with their cofactor Extradenticle (Exd) preferred sequences with two MGW minima, whereas all posterior Hox proteins in complex with Exd preferred sequences with only one MGW minimum. Interestingly, most Hox proteins share the same amino acids at key positions of their recognition helix (Supplementary Figure S8). Furthermore, most anterior and posterior Hox proteins in mouse have basic amino acids at positions 2, 3 and 5. The main difference between the two groups is position 4. Whereas almost all posterior Hox proteins exhibit a lysine at position 4 (only Hoxc9 possesses an arginine at this position), none of the anterior Hox proteins have a lysine at position 4 of the N-terminal tail. Therefore, the lysine at position 4 can explain the difference in binding specificity between the two groups of Hox TFs. The preference for a lysine at this position can also be influenced by its larger desolvation energy compared with arginine (8): an intrusion into a narrow minor groove requires desolvation, whereas a wider minor groove likely allows a lysine to retain part of its hydration shell. Homeodomains with basic amino acids, especially lysine, at position 6 of the N-terminal tail seemed to prefer sequences with wider minor groove regions at positions 4 and 5 of the binding site. These results are in agreement with previous studies, which suggested that residues at positions 6 can influence the binding specificity within the homeodomain family (16,36).

### DNA shape information improves binding affinity predictions

**Figure 5.**
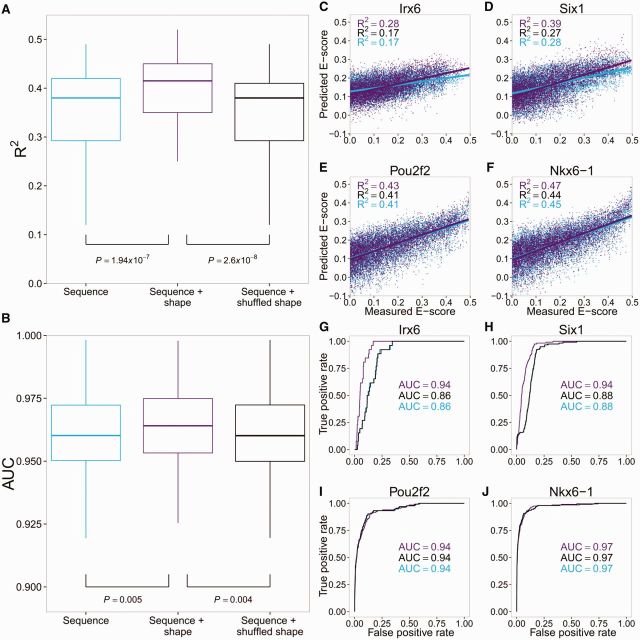
Prediction of PBM binding affinity based on a combination of DNA sequence and shape compared with DNA sequence alone. MLR was used to predict the binding affinity of each 8-mer, using the nucleotide sequences of the 8-mers (blue), the sequence and four DNA shape parameters of the 8-mers (purple) and the sequence and shuffle of the four DNA shape parameters of the 8-mers (black). (A) Box plots representing the distribution of *R*^2^ for all 168 homeodomains in mouse in the PBM data set (14). Wilcoxon *P*-values between the models indicate the significant contribution of DNA shape features. Boxes represent the median (line inside the box), 25th and 75th percentiles (edges of the box) and 5th and 95th percentiles (whiskers). (B) Box plots representing the distribution of the AUC values for all 168 homeodomains in mouse in the PBM data set (14). Wilcoxon *P*-values between the models indicate the significant contribution of DNA shape features. The boxes represent the median (line inside the box), 25th and 75th percentiles (edges of the box) and 5th and 95th percentiles (whiskers). (C–F) Correlations between experimental and predicted binding affinities for each 8-mer for the homeodomains Irx6, Six1, Pou2f2 and Nkx6-1. (G–J) ROC curves analyzing binding affinity prediction accuracies for Irx6, Six1, Pou2f2 and Nkx6-1.

Our results indicate that DNA shape information can provide a new mechanistic understanding of homeodomain–DNA recognition. Consequently we asked whether DNA shape information can contribute to the prediction accuracy of binding affinities of homeodomains to their DNA binding sites. We trained an MLR model to predict the PBM E-scores (14) for each homeodomain separately (without taking into account the amino acid sequence) as a measure of binding affinity to all possible 8-mers. The model was trained using DNA sequence and shape parameters (MGW, ProT, Roll and HelT) (24). To assess the effect of adding DNA shape information to the model, we also trained an MLR model to predict E-scores using DNA sequence alone. Using 10-fold cross validation, we showed that adding all four DNA shape features can provide additional predictive power, with a median *R*^2^ of 0.42 over all homeodomains, compared with a median *R*^2^ of 0.38 when only the DNA sequence information was considered. This improvement in binding affinity prediction accuracy of ∼10.5% was statistically significant (Mann–Whitney U *P* = 1.95 × 10^−^^7^, Figure 5A and Supplementary Table S5). This result is in agreement with our recent study on bHLH TFs in which we also showed that adding shape information improved binding affinity prediction accuracies (7).

To assess whether using one of the shape parameters is sufficient to achieve the improvement in *R*^2^, we further calculated *R*^2^ using the DNA sequence and each of the shape parameters separately. The contribution of each shape parameter was similar, reaching a median *R*^2^ of 0.39 for MGW, ProT and HelT, and 0.4 for Roll. Because adding independent variables to an MLR model generally tends to improve the *R*^2^, we predicted the E-scores again, this time shuffling the four added variables. The median *R*^2^ over all homeodomains remained at the original value of 0.38. Based on these results, we concluded that the increase in *R*^2^ was due to the additional shape information that was added to the MLR model.

We further assessed the accuracy of our DNA shape-based prediction of binding affinities and calculated the ROC curve for each homeodomain and evaluated the accuracy of our prediction in distinguishing bound (E-score ≥ 0.45) from unbound (E-score < 0.45) 8-mers. Adding the four DNA shape features increased the area under the curve (AUC) (median AUC = 0.964 over all 168 homeodomains) compared with a model based on DNA sequence information alone (median AUC = 0.958 over all 168 homeodomains). Although the increase was relatively small, it was statistically significant (Mann–Whitney U *P* = of 0.004; Figure 5B and Supplementary Table S6). This finding demonstrates that adding DNA shape information can improve the predictive power of homeodomain binding affinities to different 8-mers. In comparison, DNA sequence information with shuffled DNA shape values did not improve the prediction (median AUC = 0.958 over all 168 homeodomains; Mann–Whitney U *P* = 0.004). The AUC scores depend on the selection of a threshold defining bound and unbound probes. Although the E-score threshold, which is consistent with previous studies (14), was rather arbitrary, the AUC results complemented the results obtained using *R*^2^.

We generally observed improved binding affinity prediction accuracies on adding shape information. However, this improvement was not uniform; predictions for some homeodomains resulted in a larger improvement than for others (see Supplementary Tables S7 and S8 for coefficients and binding site logos derived from MLR models). Two proteins from the Irx and Six subfamilies demonstrated significant improvements in binding affinity prediction accuracies of 0.11 in *R*^2^, and 0.08 and 0.06 in AUC, respectively, on adding shape parameters (Figure 5C–J). Two other examples of homeodomains, Pou2f2 and Nkx6-1, yielded more subtle improvements in prediction accuracies of only 0.02 in *R*^2^ and no improvement in AUC on adding shape parameters. Overall, the effect of DNA shape information on binding affinity predictions may depend on the specific mechanisms of structural and electrostatic readout used by a given protein (2).

Until recently, the binding specificity of most TFs was modeled using the traditional definition of PWMs (18), which assumes that the nucleotide positions within a binding site contribute independently to binding affinity (19,47,48). However, several studies have shown that this assumption is an approximation that does not always hold, and there can be significant correlations between positions within the binding site (49–51). Indeed, information from dinucleotides can improve the modeling and identification of TF binding sites (52,53). We suggest that by adding DNA shape features we can implicitly account for the multiple dependencies between positions within a binding site. This concept complements observations made with dinucleotide-based models (52), but only the ability to derive DNA shape parameters in a HT manner makes this approach feasible. Moreover, the use of structural information allows shape-augmented models to be trained with only a modest increase in the number of independent variables in contrast to extended sequence features such as 2-, 3- and 4-mers (7). This aspect is important because models with fewer parameters can be trained with much smaller data sets, which can lead to additional applications.

## CONCLUSIONS

Growing evidence suggests that the sequence-dependent DNA shape plays an important role in homeodomain–DNA recognition (4,20). However, the few available experimentally solved structures of homeodomain–DNA complexes do not provide a complete understanding of the roles of individual amino acids in shape readout. To improve our understanding of the mechanisms of homeodomain–DNA recognition, we used a new HT approach for DNA shape prediction (24) and analyzed the structural feature preferences of a large set of homeodomain binding data from HT experiments (14,16). We studied the correlation between the amino acid sequences of homeodomain TFs and the nucleotide sequences and shapes of their DNA binding sites. We detected high correlation between the amino acids in the recognition helix and the sequence of the binding sites, and also between the amino acids in the N-terminal tail and the shape of the DNA binding sites. These findings suggest that the recognition helix is important for base readout and the N-terminal tail is important for shape readout, generalizing previous observations made for a small number of co-crystal structures (2,3,54). The insertion of a recognition helix into the major groove likely stabilizes intrinsic minor groove geometries (4,8), resulting in the homeodomain family of TFs using conformation capture as a predominant recognition mode.

By analyzing large sets of sequence data derived from experimental HT studies of homeodomain–DNA binding, with no available structures for most of the homeodomains, we identified amino acids that are important for shape readout. Using hypergeometric and MI tests, we found high correlations between specific amino acids at positions 2–7 of the N-terminal tail and the shape of their DNA binding sites. One drawback of MI and hypergeometric analyses is that interdependencies between residues could influence the results. For example, if there is a high interdependency between two positions—one that is important for recognition and one that is not—then both positions can appear to be important for recognition. Thus, it is not possible to determine the precise impact of individual amino acids in selecting different DNA shape features. Some proteins (including bHLH TFs) contact different base pairs in the major and the minor grooves (7). However, in homeodomain TFs, shape recognition in the minor groove is determined by the same base pairs that are recognized in the major groove through base readout, making it difficult to separate the effects of sequence and shape readout for these proteins. Nonetheless, combining our results with what is known from different experimental structures of homeodomain–DNA complexes enabled us to define specific rules, which represent a rather simple DNA shape recognition code for homeodomain TFs. We believe that our results add another important layer of information to the current understanding of homeodomain–DNA recognition.

## SUPPLEMENTARY DATA

Supplementary Data are available at NAR Online.

## FUNDING

USC-Technion Visiting Fellows Program; National Institutes of Health (NIH) [U01GM103804 and R01HG003008; in part to R.R.]; Israeli Science Foundation [1623/12 to Y.M.G.]. Funding for open access charges: USC-Technion Visiting Fellows Program.

*Conflict of interest statement*. None declared.

## Supplementary Material

Supplementary Data
