# Supplementary material for: Covariation between homeodomain transcription factors and the shape of their DNA binding sites
Source: Nucleic Acids Res. 2013 Sep 27;42(1):430–41. doi: 10.1093/nar/gkt862 (PMC3874178; doi:10.1093/nar/gkt862)
Supplement: Supplementary Data [file supp_42_1_430__index.html]

Covariation between homeodomain transcription factors and the shape of their DNA binding sites — Covariation between homeodomain transcription factors and the shape of their DNA binding sites — Supplementary Data 

# Covariation between homeodomain transcription factors and the shape of their DNA binding sites

## Supplementary Data

files

**Files in this Data Supplement:**

- Supplementary Data - pdf file
- Supplementary Data - xls file
