# Supplementary material for: Covariation between homeodomain transcription factors and the shape of their DNA binding sites
Source: Nucleic Acids Res. 2013 Sep 27;42(1):430–41. doi: 10.1093/nar/gkt862 (PMC3874178; doi:10.1093/nar/gkt862)
Supplement: Supplementary Data [file supp_gkt862_nar-01013-m-2013-File007.pdf]

SUPPLEMENTARY DATA

Covariation between homeodomain transcription factors and the shape of their DNA binding sites

Iris Dror[1,2], Tianyin Zhou[1], Yael Mandel-Gutfreund[2,*], and Remo Rohs[1,*]

[1] Molecular and Computational Biology Program, University of Southern California, Los Angeles, CA 90089, USA

[2] Department of Biology, Technion – Israel Institute of Technology, Technion City, Haifa 32000, Israel

*To whom correspondence should be addressed: Tel: +1 213 7400557, Fax: +1 213 8214257, Email: rohs@usc.edu (R.R.)

Correspondence may also be addressed to: Tel: +972 4 8293958, Fax: +972 4 8225153, Email: yaelmg@tx.technion.ac.il (Y.M.G.)

SUPPLEMENTARY FIGURES

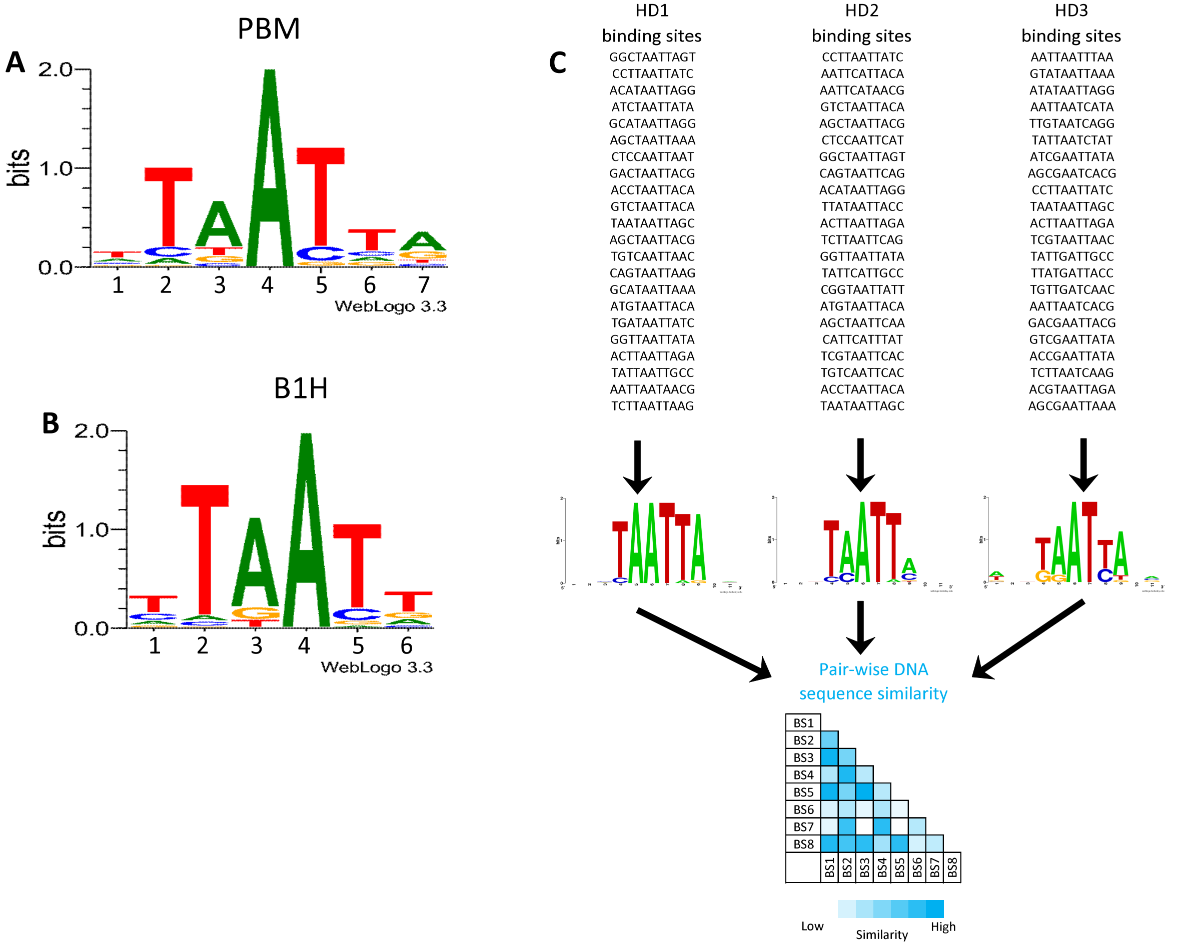

Supplementary Figure S1: (A) Binding site logo representing the DNA binding preference of all homeodomains in the mouse PBM data (14). **(B)** Binding site logo representing the DNA binding preference of all homeodomains in the *Drosophila* B1H data (16). **(C)** Flowchart for measuring the similarity between the nucleotide sequences of the homeodomain (HD)-DNA binding sites. For each HD we collected all preferred binding sequences (as explained in detail in Materials and Methods), and using these sequences, we calculated a position frequency matrix (PFM) representing the DNA binding preferences of each HD. Next, we compared pairs of PFMs using Pearson Correlation Coefficient (PCC) to derive the pair-wise DNA sequence similarity.

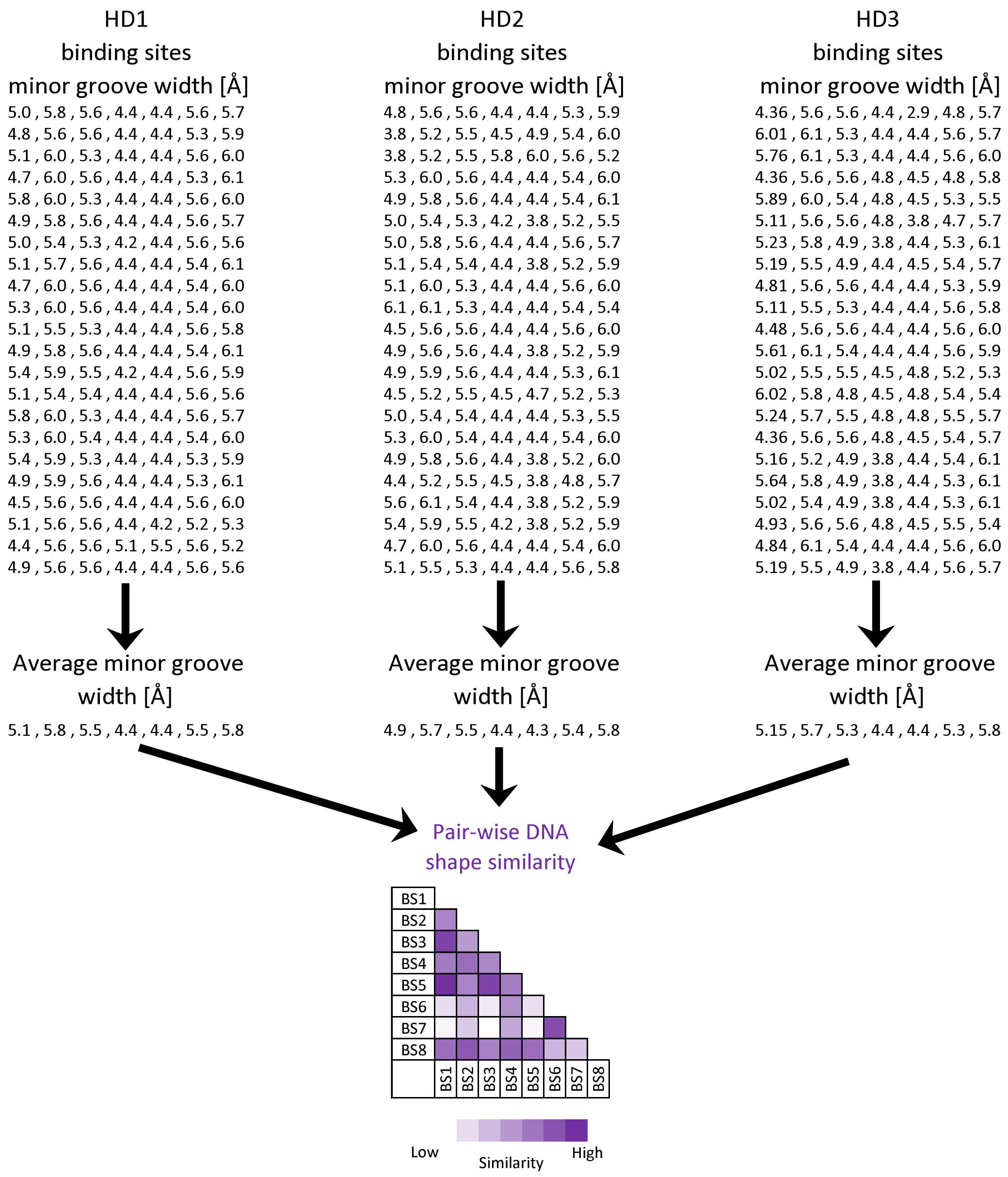

Supplementary Figure S2: Flowchart for measuring the similarity between the DNA shapes of the HD binding sites. For each HD we collected all preferred DNA binding sequences (see Materials and Methods for details), and using these sequences, we predicted the different shape parameters for each nucleotide position and calculated an average shape preference for each HD. Next, we compared pairs of average shape preferences using Euclidian distance to derive the pair-wise DNA shape similarity.

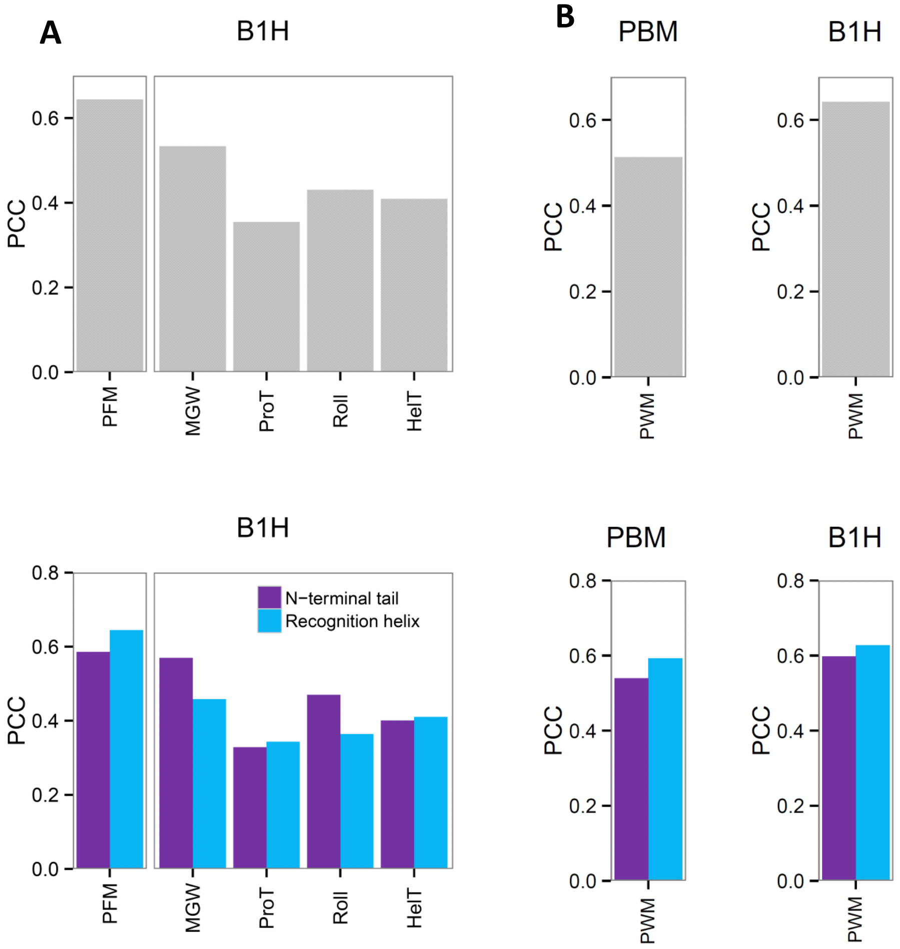

Supplementary Figure S3: (A) Plots representing the correlation between the pair-wise HD sequence similarity and DNA binding site similarities, based on five features (nucleotide sequence (PFM) and the four DNA shape parameters minor groove width (MGW), propeller twist (ProT), Roll, and Helix Twist (HelT)), using B1H data (16). **(B)** Plots representing the correlation between the pairwise HD sequence similarity and DNA binding site sequences using position weight matrices (PWM) to describe nucleotide sequence.

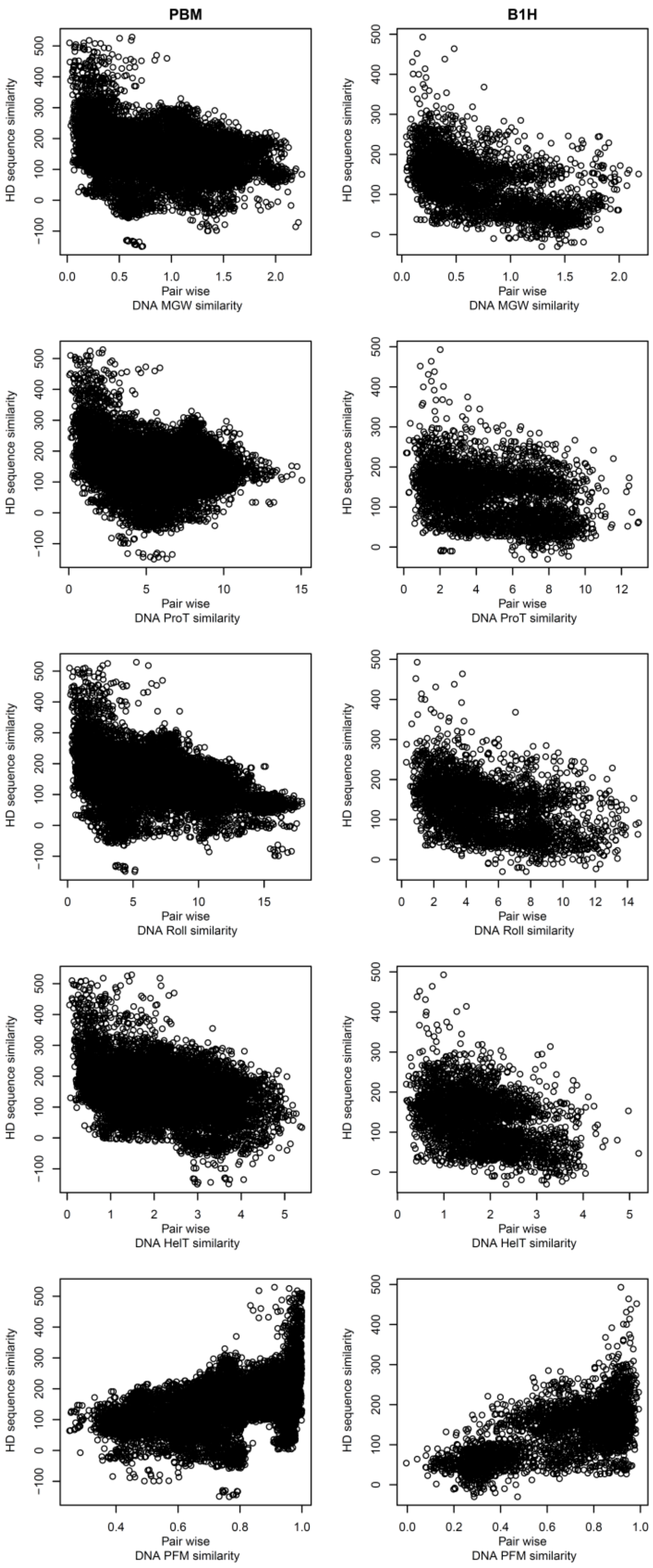

Supplementary Figure S4: Dependencies between the amino sequences of HDs and the nucleotide sequence (PFM) and shape features (MGW, ProT, Roll, and HelT) of their DNA binding sites derived from PBM (14) and B1H (16) data.

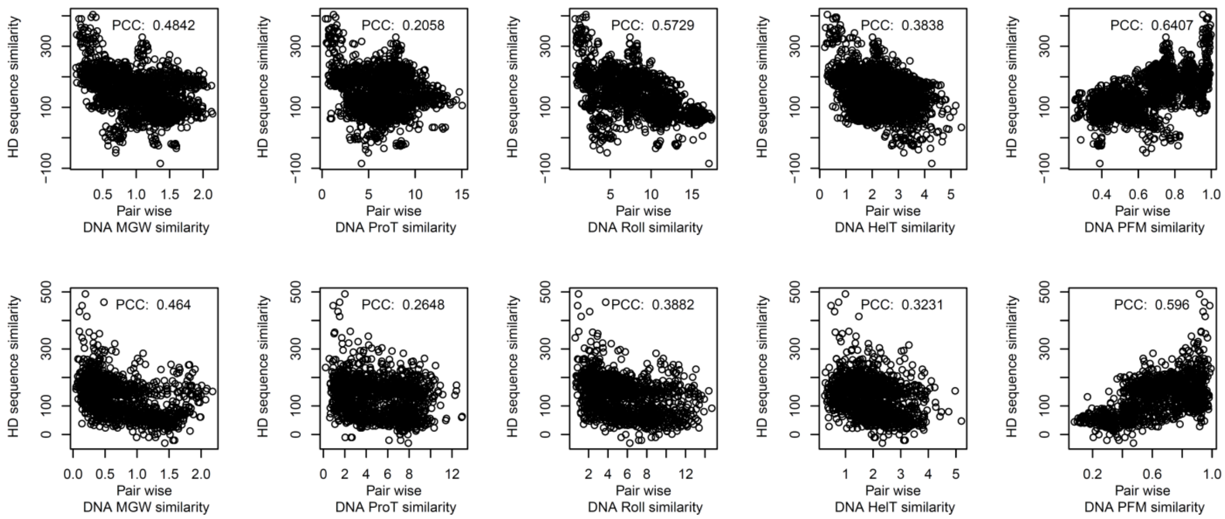

Supplementary Figure S5: Dependencies between the amino sequences of HDs and the nucleotide sequences (PFM) and shape features (MGW, ProT, Roll, and HelT) of their DNA binding sites derived from PBM (14) and B1H (16) data. Assigning the DNA PFM scores to seven bins with an equal number of binding sites (see Materials and Methods for details), we calculated the PCC for the PBM (top panel) and B1H (bottom panel). The correlations indicate that our findings are equivalent to the analysis without using bins (see also Figure 2A; Supplementary Figures S3 and S4).

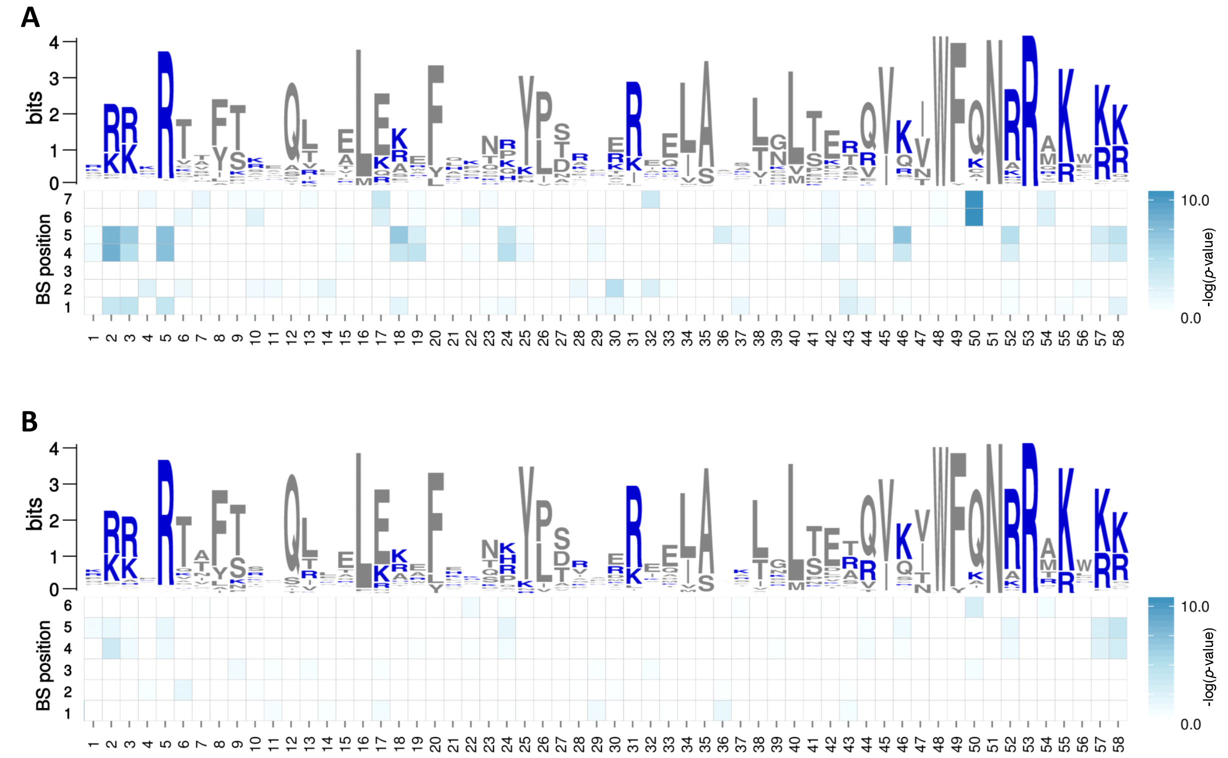

Supplementary Figure S6: Dependencies between the occurrence of positively charged amino acids in HDs and a narrow minor groove region in their DNA binding sites, based on PBM (14) and B1H (16) data. **(A)** Top: Sequence logo representing the alignment of all 168 HD sequences in mouse. The amino acids are numbered to correspond with the convention for Hoxa9 in mouse (32). Positively charged amino acids are colored in blue. Bottom: –log *p*-value of the hypergeometric score between positively charged amino acids and a narrow minor groove at each position of their preferred DNA binding sites. **(B)** Top: Sequence logo representing the alignment of all 83 HD sequences in *Drosophila*. The amino acids are numbered to correspond with the convention for Engrailed in *Drosophila* (29). Positively charged amino acids are colored in blue. Bottom: –log *p*-value of the hypergeometric score between positively charged amino acids and a narrow minor groove at each position of their preferred DNA binding sites.

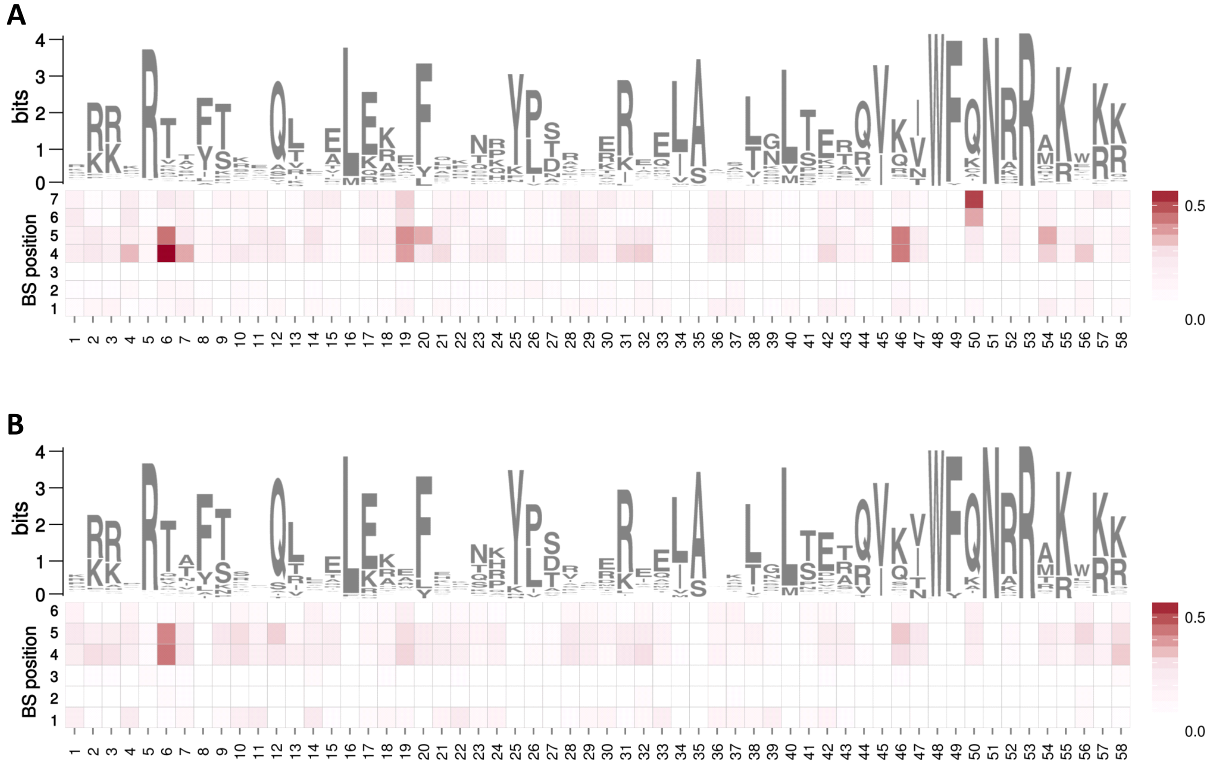

Supplementary Figure S7: Mutual information (MI) scores between each amino acid position in the HDs and a narrow minor groove region at each nucleotide position of their preferred DNA binding sites are shown for the PBM (14) and B1H (16) data. **(A)** Top: Sequence logo representing the alignment of all 168 HD sequences in mouse. The amino acids are numbered to correspond with the convention for Hoxa9 in mouse (32). Bottom: MI score between amino acids and MGW at each position of their preferred DNA binding sites. **(B)** Top: Sequence logo representing the alignment of all 83 HD sequences in *Drosophila*. The amino acids are numbered to correspond with the convention for Engrailed in *Drosophila* (29). Bottom: MI score between amino acids and MGW at each position of their preferred DNA binding sites.

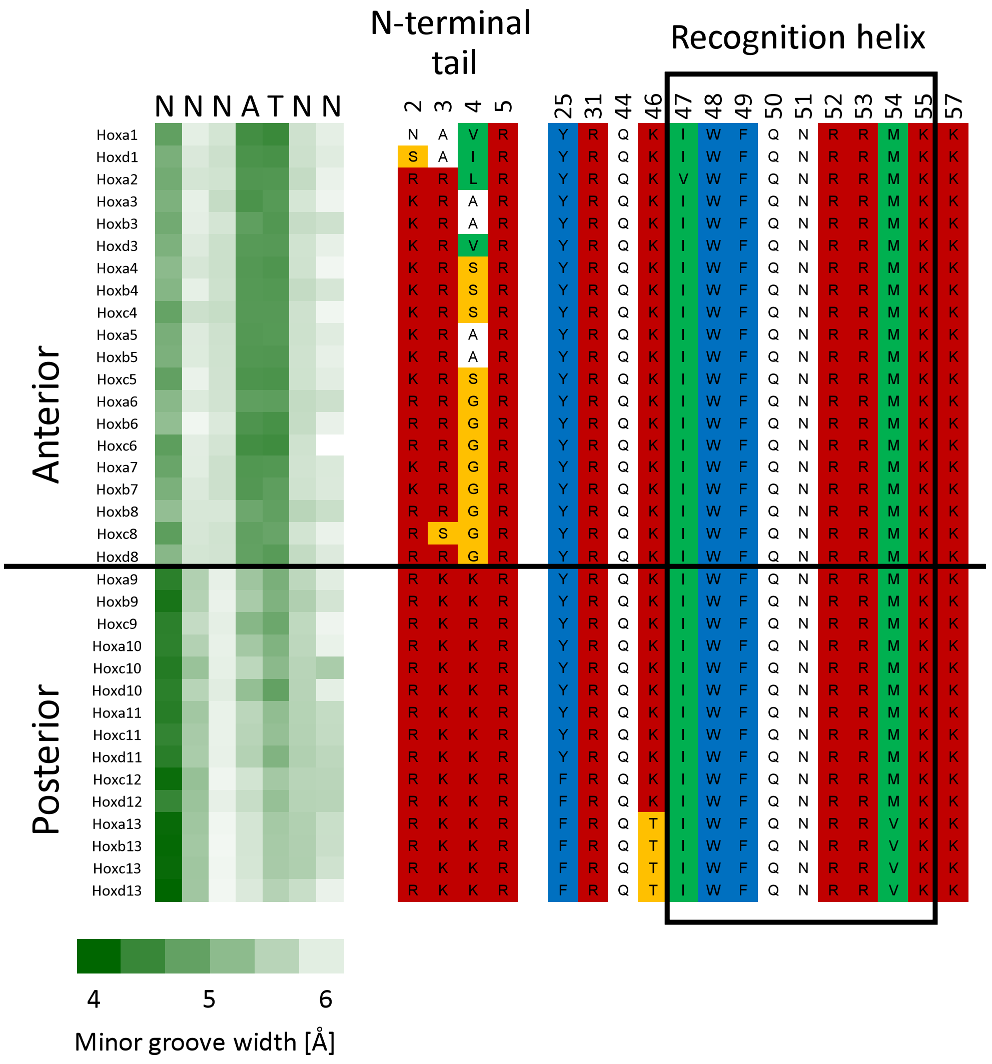

Supplementary Figure S8: DNA shape analysis of the PBM data for Hox proteins in mouse (14). Left: Average minor groove width in each position of the preferred DNA binding sites of anterior and posterior Hox proteins. Right: Variations in amino acids at the N-terminal tail, recognition helix (marked in black box), and other positions that were previously suggested to be important for HD-DNA recognition. The amino acids are colored according to the Clustal default color scheme. Note that the different DNA shape preferences of anterior and posterior Hox protein observed here for mouse have been previously discovered for *Drosophila* (20).

SUPPLEMENTARY TABLES

Supplementary Table S1: Hypergeometric test scores for the PBM dataset (14).

BS position

HD position

	1	2	3	4	5	6	7
1	0.292945	0.04992	0	1.345023	0.945645	0.001709	0.123923
2	4.018248	0.018882	0	8.475893	8.192503	0.017978	0.003874
3	4.355083	0.227073	0	4.997744	6.434828	0.002812	0.026844
4	0.009799	2.562664	0	2.14E-12	7.14E-10	0.226981	1.416225
5	4.263259	0.04696	0	7.377116	7.288451	0.014188	4.74E-05
6	5.47E-07	1.550741	0	6.33E-11	1.40E-09	1.725337	0.189629
7	0.50042	0	0	0.220468	0.09276	0.755584	1.777074
8	0	0	0	0	0	0	0
9	0.001047	0.287374	0	0.001381	0.001022	0.076653	0.990819
10	0.364762	1.283032	0	1.13E-06	4.82E-05	2.311224	0.752252
11	0.002696	1.31483	0	0.000166	0.008443	0.037322	0.62321
12	0	0	0	0	0	0	0
13	0.002114	0.772654	0	0.0231	0.063888	0.136972	1.048374
14	0.684135	1.98841	0	0.004405	0.010705	0.108294	0.326161
15	0.406873	0	0	0.91817	0.614063	0	0
16	0	0	0	0	0	0	0
17	0.000119	0.043211	0	3.79E-08	2.46E-11	2.653262	3.937763
18	2.081683	0.202525	0	4.106282	6.775098	0.000106	3.91E-06
19	0.343165	0	0	4.44158	2.955953	0.900153	0.890647
20	0	0	0	0	0	0	0
21	0.024489	0.090719	0	0.117699	0.099601	0.777717	0.030829
22	6.24E-05	0	0	1.053427	0.255646	1.748101	0.111807
23	0.788398	0	0	0.04773	0.00825	0.03272	0.961821
24	1.339748	0.174711	0	4.967307	4.046083	0.018823	0.000779
25	0.091048	0	0	1.671752	1.116955	0	0.02432
26	0	0	0	0	0	0	0
27	0	0	0	0	0	0	0
28	0.413973	1.586783	0	0.006715	0.058483	0.292311	0.000624
29	1.186053	0.149478	0	1.34859	1.372217	0.322081	0.336292
30	1.496648	4.904932	0	0.001124	0.087793	0.048015	0.299756
31	0.686776	0.684491	0	0	0	0.003767	0.00284
32	0.057358	2.794307	0	5.81E-12	2.66E-12	0.096124	3.628445
33	0.234559	0.853344	0	0.194348	0.11758	0.057072	0.032315
34	0	0	0	0	0	0	0
35	0	0	0	0	0	0	0

36	0.163298	0.158863	0	0.220455	2.955953	0.005996	0.002133
37	1.561751	0.241874	0	1.19614	1.477872	0.345505	0.034178
38	0	0	0	0	0	0	0
39	0.000935	0	0	0.009773	0.002773	1.693236	0.038163
40	0	0	0	0	0	0	0
41	0.314497	0	0	0.7094	0.474565	0.612503	0.510752
42	0.019041	0	0	2.348082	1.567406	1.251854	1.4251
43	2.801963	2.05099	0	0.123191	1.138948	0.013897	0.004195
44	1.726653	0	0	2.025447	1.077089	1.145551	0.799238
45	0	0	0	0	0	0	0
46	0.773543	0.182844	0	3.733836	7.076812	0.005116	0.012859
47	0	0	0	0	0	0	0
48	0.044116	0	0	0.099385	0.066536	0.865301	0.798355
49	0	0	0	0	0	0	0
50	0.027326	0.108653	0	0.09166	0.045108	10.74025	10.61409
51	0	0	0	0	0	0	0
52	0.856346	0.835363	0	2.839861	4.953009	2.49E-07	3.37E-06
53	0	0.025405	0	0	0	0	0
54	0.000636	0	0	8.17E-10	1.10E-12	2.731675	2.287804
55	0.980147	0.154706	0	0	0	0.387994	0.458672
56	0.406873	1.072702	0	0.137257	0.203551	0	0
57	0	0.342453	0	2.234064	3.304592	0.036994	3.83E-06
58	2.208479	0.277556	0	3.417947	4.657535	0.001515	5.34E-08

Supplementary Table S2: Hypergeometric test scores for the B1H dataset (16).

BS position

HD position

	1	2	3	4	5	6
1	0.201363	0.06936	0.062255	0.227434	0.276018	0.169757
2	0.076128	0.013744	0.0803	0.301678	0.210324	0.032724
3	0.045843	0.028535	0.0803	0.295216	0.226871	0.030147
4	0.24368	0.06936	0.049261	2.25E-01	2.41E-01	0.154102
5	0.012281	0.000936	0.109286	0.097194	0.110724	7.91E-03
6	8.67E-02	0.109286	0.0803	4.49E-01	4.26E-01	0.086957
7	0.130805	0.0803	0.0803	0.278362	0.26686	0.114818
8	0.030932	0.041315	0.006374	0.049437	0.04934	0.026716
9	0.107354	0.028535	0.062255	0.231639	0.287507	0.057272
10	0.213416	0.056972	0.056972	2.96E-01	3.04E-01	0.146179
11	0.243111	0.056972	0.052762	0.202011	0.181016	0.102667
12	0.056549	0.001917	0.062255	0.265404	0.31886	0.016223
13	0.040888	0.046264	0.043643	0.214399	0.152637	0.016614
14	0.258535	0.032468	0.109286	0.225799	0.197379	0.175828
15	0.08614	0.016243	0.062255	0.225118	0.226131	0.112173
16	0.012281	0.000936	0.000936	0.007914	0.00584	0.007914
17	0.100655	0.005562	0.06936	1.09E-01	1.55E-01	0.09723
18	0.143508	0.023279	0.0803	0.180384	0.1451	1.11E-01
19	0.122976	0.026272	0.062255	0.312731	0.303289	0.208575
20	0.038815	0.002945	0.052762	0.247285	0.240036	0.024966
21	0.169402	0.046264	0.052762	0.221464	0.21912	0.10374
22	2.55E-01	0.052762	0.0803	0.218841	0.190509	0.11061
23	0.132606	0.02237	0.02237	0.127701	0.169434	0.106513
24	0.164102	0.062255	0.046264	0.176922	0.209814	0.083185
25	0.016599	0.002945	0.002945	0.024966	0.018406	0.036682
26	0.040743	0.062255	0.017615	0.117575	0.09658	0.017292
27	0.059217	0.023279	0.023279	0.10435	0.096225	0.023877
28	0.086826	0.062255	0.056972	0.289302	0.268913	0.132451
29	0.144141	0.06936	0.046264	0.210998	0.218654	0.138483
30	0.1501	0.062255	0.027373	0.127862	0.174745	0.101993
31	0.08651	0.0044	0.049261	0.277153	0.23378	0.078971
32	0.129124	0.109286	0.06936	3.00E-01	2.48E-01	0.12646
33	0.212537	0.016243	0.062255	0.152096	0.171155	0.092523
34	0.017183	0.009494	0.009494	0.081384	0.059794	0.024126
35	0.009572	0.00366	0.00366	0.009618	0.017268	0.002979
36	0.166488	0.062255	0.062255	0.159757	0.141422	0.156156
37	0.154607	0.0803	0.049261	0.113695	0.144507	0.164476

38	0.146125	0.008554	0.035573	0.138871	0.178663	0.020985
39	0.207143	0.06936	0.026272	0.106101	0.100488	0.096019
40	0.008736	0.001258	0.001258	0.010638	0.007849	0.010638
41	0.104106	0.016918	0.016918	0.088344	0.107766	0.105364
42	0.160555	0.009494	0.0803	0.197261	0.22854	0.127039
43	0.079791	0.028535	0.031074	0.182042	0.219434	0.095734
44	0.04169	0.010478	0.010478	0.089994	0.06608	0.046784
45	0.013004	0.006793	0.006793	0.057938	0.042634	0.014701
46	0.061389	0.013163	0.0803	0.299885	0.330106	0.13191
47	0.060511	0.021499	0.052762	0.2223	0.281918	0.004569
48	0.004027	0.000307	0.000307	0.002597	0.001917	0.046264
49	0.00812	0.00062	0.00062	0.005234	0.003863	0.019567
50	0.052585	0.00366	0.052762	0.190873	0.240751	0.190873
51	0	0	0	0	0	0
52	0.053219	0.004027	0.004027	0.092989	0.110132	2.13E-02
53	0	0	0	0	0	0
54	0.069108	0.033963	0.06936	1.96E-01	2.41E-01	0.073362
55	0.038815	0.002945	0.043643	0.139271	0.190617	1.01E-02
56	0.118337	0.109286	0.056972	0.257677	0.308839	0.173313
57	0.016513	0.012598	0.019858	0.184083	0.180475	0.0533
58	0.074614	0.014341	0.109286	0.328309	0.302299	0.141612

Supplementary Table S3: Mutual information scores for the PBM dataset (14).

BS position

HD position

	1	2	3	4	5	6	7
1	0.094798	0.081373	0	0.237079	0.2082	0.257948	0.186747
2	0.146119	0.08228	0	0.278428	0.256386	0.099587	0.089062
3	0.169107	0.082349	0	0.223504	0.194932	0.061654	0.098035
4	0.074743	0.125748	0	3.53E-01	2.17E-01	0.26922	0.236924
5	0.099804	0.012717	0	0.141728	0.158962	7.51E-02	8.96E-02
6	1.24E-01	0.145494	0	5.79E-01	4.55E-01	0.123428	0.250919
7	0.171951	0.136851	0	0.378239	0.259009	0.190008	0.20654
8	0.129064	0.076084	0	0.142945	0.144532	0.104217	0.04834
9	0.0528	0.021674	0	0.170449	0.184164	0.040914	0.149877
10	0.120761	0.111179	0	2.73E-01	1.74E-01	0.104002	0.153556
11	0.094376	0.10573	0	0.212553	0.252122	0.143094	0.145597
12	0.139161	0.023524	0	0.231241	0.253425	0.047233	0.062205
13	0.063686	0.040581	0	0.14673	0.091636	0.094335	0.09359
14	0.127254	0.084786	0	0.241037	0.293128	0.157094	0.220367
15	0.106513	0.062327	0	0.12412	0.132414	0.138033	0.231319
16	0.011269	0.006483	0	0.018785	0.030043	0.003411	0.026247
17	0.074742	0.024395	0	1.69E-01	2.37E-01	0.091336	0.195679
18	0.090354	0.069414	0	0.152875	0.192684	1.16E-01	1.63E-01
19	0.177207	0.086638	0	0.39338	0.413623	0.302549	0.32149
20	0.056872	0.033511	0	0.277107	0.373347	0.056239	0.051989
21	0.138821	0.0774	0	0.304559	0.263156	0.181246	0.144045
22	9.85E-02	0.085183	0	0.111122	0.128268	0.172014	0.159729
23	0.077289	0.085631	0	0.236415	0.184471	0.110428	0.170551
24	0.09744	0.093434	0	0.247264	0.221795	0.077973	0.121719
25	0.05316	0.102361	0	0.103343	0.079615	0.042799	0.032972
26	0.031039	0.142306	0	0.151603	0.122594	0.115292	0.141936
27	0.056946	0.095133	0	0.13075	0.065144	0.085692	0.124197
28	0.125986	0.069476	0	0.256187	0.240377	0.202664	0.248864
29	0.159084	0.079057	0	0.295323	0.243316	0.202109	0.152744
30	0.111857	0.10258	0	0.162315	0.157647	0.1176	0.22601
31	0.102144	0.018489	0	0.313248	0.25687	0.085909	0.127579
32	0.142541	0.133907	0	3.23E-01	2.89E-01	0.096029	0.154785
33	0.129229	0.055182	0	0.102289	0.120625	0.083865	0.061125
34	0.017056	0.013697	0	0.01761	0.022566	0.017538	0.014431
35	0.02452	0.075549	0	0.06981	0.020708	0.015124	0.010272
36	0.180147	0.068223	0	0.2479	0.275945	0.259677	0.203744
37	0.178621	0.120676	0	0.23094	0.239813	0.232774	0.156269

38	0.042501	0.0126	0	0.119413	0.208781	0.174124	0.144872
39	0.079679	0.053268	0	0.108704	0.084834	0.088187	0.101889
40	0.125757	0.017582	0	0.026208	0.03665	0.098834	0.084169
41	0.050128	0.048161	0	0.085642	0.057492	0.068306	0.039538
42	0.195425	0.061075	0	0.30772	0.257441	0.18304	0.162056
43	0.152532	0.08059	0	0.187167	0.191834	0.099448	0.172982
44	0.048258	0.044824	0	0.169437	0.116412	0.171576	0.16394
45	0.022376	0.026355	0	0.027912	0.009141	0.005592	0.014837
46	0.18448	0.099897	0	0.441234	0.440735	0.103766	0.177235
47	0.124106	0.055107	0	0.240996	0.238123	0.03436	0.112228
48	0.000835	0.000481	0	0.001882	0.00126	0.016482	0.015193
49	0.018739	0.002433	0	0.009532	0.006378	0.02116	0.020757
50	0.121299	0.020986	0	0.213039	0.262806	0.379491	0.520845
51	0.001675	0.000965	0	0.003776	0.002527	0.017641	0.016571
52	0.051783	0.025696	0	0.172312	0.20955	1.53E-01	1.44E-01
53	0.000835	0.000481	0	0.001882	0.00126	0.016482	0.015193
54	0.212176	0.074962	0	3.19E-01	3.74E-01	0.181977	0.186228
55	0.104841	0.004926	0	0.166562	0.196252	3.37E-02	0.058791
56	0.150544	0.144287	0	0.330128	0.252681	0.118517	0.140134
57	0.012351	0.006968	0	0.105343	0.125935	0.075185	2.41E-01
58	0.104512	0.022007	0	0.175824	0.19423	0.072543	1.57E-01

Supplementary Table S4: Mutual information scores for the B1H dataset (16).

BS position

HD position

	1	2	3	4	5	6
1	0.423016	0	0	0.335491	1.107145	0.013769
2	0	0.053519	0	3.606906	1.746909	0.453222
3	0	0.053519	0	1.352032	0.854491	0.453222
4	0.352139	0.838849	0	4.45E-04	5.46E-05	0.133832
5	0	0.019305	0	1.472783	1.734754	1.63E-01
6	8.30E-02	1.838849	0	0.00E+00	0.00E+00	0
7	0.253007	0	0	0.1631	0.120371	0.504332
8	0	0	0	0	0	0
9	0.340157	0	1.236789	0.001755	0.000641	0
10	0.362453	0.634729	0	5.96E-03	8.69E-04	0.222544
11	0.994962	0	0.797456	0.050105	0.02102	0.113082
12	0	0	0	0	0	0
13	0.293594	0	0.884607	0.000232	0.011398	0.158642
14	0.695087	0	0	0.027536	0.051506	0.057712
15	0.006408	0	0	0.393224	0.290042	0
16	0	0	0	0	0	0
17	1.198294	0	0.724906	1.42E-04	6.22E-06	0
18	0.032899	0.2156	0	0.4191	0.359603	6.86E-02
19	0.116214	0	0	0.576424	0.424971	0.485724
20	0	0	0	0	0	0
21	0.244917	0	0	0.210307	0.098605	0.149464
22	2.71E-04	0	0.51663	0.638176	0.395421	1.300119
23	0.099992	0	0	0.045357	0.025287	0
24	0.500435	0	0	1.209634	1.960701	0.692326
25	0.187978	0	0	0.393224	0.290042	0.225021
26	0	0	0	0	0	0
27	0	0	0	0	0	0
28	0.327359	0.51663	0	0.269014	0.395421	0.113538
29	1.303029	0	0.692721	0.096426	0.188359	0.068347
30	0.284508	0.537819	0	0.238702	0.361721	0.130418
31	0.156587	0.039509	0.039509	0	0	0.007849
32	0.293594	0	0.884607	2.32E-04	8.36E-04	0
33	0.518972	0	0	0.065401	0.246577	0
34	0	0	0	0	0	0
35	0	0	0	0	0	0
36	1.744503	0.583577	0	0.183805	0.085181	0.170946
37	0.004639	0	0	0.335491	0.467076	0.605883

38	0	0	0	0	0	0
39	0.148217	0	0	0.639706	0.471547	0
40	0	0	0	0	0	0
41	0.253007	0	0	0.1631	0.120371	0.504332
42	0.034329	0	0	0.1631	0.120371	0
43	1.025562	0.560095	0	0.210307	0.32941	0.028821
44	0.01553	0	0	1.045753	0.769991	0.513056
45	0	0	0	0	0	0
46	0.199169	0.236789	0	0.715311	1.360633	0.261387
47	0	0	0	0	0	0
48	0	0	0	0	0	0
49	0	0	0	0	0	0
50	0.141553	0	1.060698	0.007849	0.002995	2.959667
51	0	0	0	0	0	0
52	0	0.053519	0.053519	1.352032	1.746909	1.14E-01
53	0	0	0	0	0	0
54	0.428795	0	0	1.67E-04	3.68E-05	1.003619
55	0	0	0	0	0	0.00E+00
56	0.063951	0	0	0.219216	0.161763	0.40192
57	0	0.025936	0.025936	2.394438	2.831255	0.028401
58	0.075073	0.060698	0	3.272697	4.195417	0.003437

Supplementary Table S5: *R^2* values for E-score predictions for each of the 168 HDs in mouse (14) using multiple linear regression (MLR).

HD	Sequence	Sequence + shape	Sequence + shuffled shape
Alx3_3418.2	0.46	0.49	0.46
Alx4_1744.1	0.39	0.42	0.39
Arx_1738.2	0.43	0.48	0.43
Bapx1_2343.1	0.29	0.35	0.29
Barhl1_2590.2	0.35	0.39	0.35
Barhl2_3868.1	0.38	0.42	0.38
Barx1_2877.1	0.3	0.36	0.3
Barx2_3447.2	0.35	0.41	0.34
Bsx_3483.2	0.34	0.4	0.34
Cart1_0997.1	0.43	0.47	0.43
Cart1_1275.1	0.33	0.36	0.32
Cdx1_2245.1	0.42	0.44	0.41
Cdx2_4272.1	0.42	0.45	0.42
Cphx_3484.1	0.23	0.26	0.23
Crx_3485.1	0.27	0.33	0.27
Cutl1_3494.1	0.33	0.35	0.33
Cutl1_3494.2	0.32	0.34	0.32
Dbx1_3486.1	0.42	0.45	0.41
Dbx2_3487.1	0.46	0.49	0.46
Dlx1_1741.2	0.45	0.49	0.45
Dlx2_2273.2	0.41	0.45	0.4
Dlx3_1030.1	0.42	0.45	0.42
Dlx4_3488.2	0.43	0.46	0.43
Dlx5_3419.2	0.42	0.46	0.42
Dmbx1_2277.1	0.28	0.33	0.28
Dobox4_3956.2	0.24	0.26	0.24
Dobox5_3493.1	0.28	0.32	0.28
Duxl_1286.2	0.34	0.39	0.34
Emx2_3420.1	0.38	0.42	0.38
En1_3123.2	0.44	0.46	0.43
En2_0952.1	0.44	0.47	0.44
Esx1_3124.2	0.48	0.52	0.48
Evx1_3952.2	0.37	0.42	0.37
Evx2_2645.3	0.37	0.42	0.36
Gbx1_2883.2	0.41	0.44	0.4
Gbx2_3110.1	0.4	0.44	0.4

Gsc_2327.3	0.32	0.37	0.31
Gsh2_3990.2	0.39	0.41	0.39
Hdx_3845.3	0.27	0.3	0.27
Hlx1_2350.1	0.42	0.44	0.41
Hlxb9_3422.1	0.43	0.47	0.42
Hmbox1_2674.1	0.33	0.37	0.33
Hmx1_3423.1	0.4	0.43	0.39
Hmx2_3424.3	0.38	0.42	0.38
Hmx3_3490.2	0.41	0.45	0.41
Homez_1063.2	0.12	0.16	0.12
Hoxa1_3425.1	0.31	0.33	0.31
Hoxa10_2318.1	0.43	0.46	0.43
Hoxa11_2218.1	0.41	0.44	0.4
Hoxa13_3126.1	0.42	0.46	0.42
Hoxa2_3079.1	0.4	0.44	0.4
Hoxa3_2783.2	0.41	0.46	0.4
Hoxa4_3426.1	0.38	0.4	0.37
Hoxa5_3415.1	0.4	0.44	0.4
Hoxa6_1040.1	0.38	0.41	0.38
Hoxa7_2668.2	0.4	0.43	0.4
Hoxa7_3750.1	0.36	0.38	0.35
Hoxa9_2622.2	0.44	0.48	0.44
Hoxb13_3479.1	0.44	0.48	0.43
Hoxb3_1720.2	0.36	0.4	0.36
Hoxb4_2627.1	0.4	0.43	0.39
Hoxb5_3122.2	0.39	0.42	0.39
Hoxb6_3428.2	0.34	0.37	0.34
Hoxb7_3953.1	0.42	0.44	0.41
Hoxb8_3780.2	0.43	0.46	0.43
Hoxb9_3413.1	0.4	0.44	0.4
Hoxc10_2779.2	0.33	0.36	0.33
Hoxc11_3718.2	0.34	0.38	0.34
Hoxc12_3480.1	0.36	0.41	0.36
Hoxc13_3127.1	0.43	0.47	0.43
Hoxc4_3491.1	0.38	0.41	0.38
Hoxc5_2630.2	0.37	0.4	0.37
Hoxc6_3954.2	0.39	0.41	0.39
Hoxc8_3429.2	0.4	0.43	0.4
Hoxc9_2367.2	0.4	0.43	0.4
Hoxd1_3448.1	0.37	0.41	0.36
Hoxd10_2368.2	0.48	0.5	0.47

Hoxd11_3873.1	0.38	0.42	0.38
Hoxd12_3481.1	0.4	0.44	0.4
Hoxd13_2356.1	0.42	0.47	0.42
Hoxd3_1742.2	0.38	0.42	0.38
Hoxd8_2644.1	0.39	0.42	0.39
Ipf1_3815.1	0.39	0.43	0.39
Irx2_0900.3	0.15	0.27	0.15
Irx3_0920.1	0.15	0.25	0.15
Irx3_2226.1	0.16	0.26	0.16
Irx4_2242.3	0.17	0.27	0.17
Irx5_2385.1	0.16	0.26	0.16
Irx6_2623.2	0.17	0.28	0.17
Isl2_3430.1	0.32	0.38	0.32
Isx_3445.1	0.41	0.45	0.4
Lbx2_3869.2	0.36	0.38	0.35
Lhx1_2240.2	0.46	0.49	0.46
Lhx2_0953.2	0.4	0.43	0.39
Lhx3_3431.1	0.43	0.47	0.43
Lhx4_1719.2	0.41	0.45	0.41
Lhx5_2279.1	0.44	0.47	0.44
Lhx6_2272.1	0.41	0.45	0.41
Lhx6_3432.1	0.37	0.4	0.37
Lhx8_2247.2	0.4	0.43	0.39
Lhx9_3492.1	0.41	0.44	0.41
Lmx1a_2238.2	0.49	0.51	0.49
Lmx1b_3433.2	0.45	0.47	0.45
Meis1_2335.1	0.19	0.26	0.19
Meox1_2310.2	0.39	0.44	0.39
Mrg1_2246.2	0.2	0.27	0.2
Mrg2_2302.1	0.22	0.3	0.22
Msx1_3031.2	0.35	0.38	0.34
Msx2_3449.1	0.42	0.47	0.42
Msx3_3206.1	0.48	0.52	0.48
Nkx1-1_3856.3	0.34	0.38	0.33
Nkx1-2_3214.1	0.38	0.42	0.38
Nkx2-2_2823.1	0.2	0.27	0.2
Nkx2-3_3435.1	0.26	0.31	0.26
Nkx2-4_3074.1	0.21	0.29	0.21
Nkx2-5_3436.1	0.23	0.3	0.23
Nkx2-6_3437.1	0.25	0.31	0.25
Nkx2-9_3082.1	0.2	0.25	0.19

Nkx3-1_2923.2	0.28	0.33	0.28
Nkx6-1_2825.1	0.45	0.47	0.44
Nkx6-1_2825.2	0.44	0.47	0.44
Nkx6-3_3446.1	0.47	0.49	0.46
Obox1_3970.2	0.28	0.33	0.28
Obox2_3438.2	0.3	0.32	0.3
Obox3_3439.1	0.26	0.29	0.25
Obox5_2284.1	0.29	0.31	0.29
Obox5_3963.2	0.31	0.33	0.3
Obox6_3440.2	0.31	0.36	0.31
Og2x_3719.1	0.43	0.46	0.43
Otp_3496.1	0.43	0.45	0.43
Otx1_2325.1	0.29	0.34	0.29
Otx2_3441.1	0.28	0.33	0.28
Pax4_3989.2	0.39	0.43	0.39
Pax6_3838.3	0.31	0.35	0.3
Pax7_3783.1	0.42	0.46	0.41
Pbx1_3203.1	0.28	0.36	0.28
Phox2a_3947.1	0.42	0.46	0.42
Phox2b_3948.1	0.42	0.45	0.42
Pitx1_2312.1	0.27	0.32	0.26
Pitx2_2274.3	0.26	0.31	0.26
Pitx3_3497.2	0.26	0.3	0.25
Pknox1_2364.2	0.22	0.3	0.22
Pknox2_3077.2	0.22	0.29	0.22
Pou1f1_3818.1	0.39	0.41	0.38
Pou2f1_3081.2	0.39	0.42	0.39
Pou2f2_3748.1	0.41	0.43	0.41
Pou2f3_3986.2	0.38	0.4	0.38
Pou3f1_3819.1	0.38	0.42	0.38
Pou3f2_2824.1	0.45	0.48	0.45
Pou3f3_3235.2	0.34	0.37	0.34
Pou3f4_3773.1	0.39	0.41	0.38
Pou4f3_2791.1	0.41	0.43	0.4
Pou6f1_1731.2	0.43	0.46	0.43
Pou6f1_3733.1	0.42	0.44	0.41
Prop1_3949.1	0.43	0.45	0.42
Prrx1_3442.1	0.42	0.45	0.42
Prrx2_3072.1	0.44	0.47	0.44
Rax_3443.1	0.38	0.42	0.38
Rhox11_1765.2	0.32	0.4	0.31

Rhox11_2205.1	0.3	0.38	0.3
Rhox6_4251.1	0.37	0.4	0.37
Shox2_2641.2	0.45	0.49	0.45
Six1_0935.2	0.28	0.39	0.27
Six2_2307.2	0.27	0.37	0.27
Six3_1732.2	0.28	0.38	0.28
Six4_2860.1	0.21	0.26	0.21
Six6_2267.4	0.29	0.39	0.29
Six6_2267.5	0.27	0.33	0.27
Tcf1_2666.2	0.31	0.36	0.31
Tcf2_0913.2	0.29	0.32	0.28
Tgif1_2342.2	0.2	0.27	0.19
Tgif2_3451.1	0.17	0.25	0.17
Titf1_1722.2	0.22	0.28	0.22
Tlx2_3498.2	0.36	0.39	0.35
Uncx4.1_2281.2	0.45	0.48	0.45
Vax1_3499.1	0.4	0.43	0.39
Vax2_3500.1	0.39	0.41	0.38
Vsx1_1728.1	0.44	0.47	0.43

Supplementary Table S6: AUC for E-score predictions for each of the 168 HDs in mouse (14).

HD	Sequence	Sequence + shape	Sequence + shuffled shape
Alx3_3418.2	0.955498	0.957916	0.955512
Alx4_1744.1	0.956072	0.960697	0.956244
Arx_1738.2	0.960326	0.960994	0.960374
Bapx1_2343.1	0.97637	0.976108	0.976255
Barhl1_2590.2	0.889294	0.897671	0.889121
Barhl2_3868.1	0.89054	0.909044	0.890513
Barx1_2877.1	0.912219	0.940121	0.912043
Barx2_3447.2	0.909373	0.939451	0.908992
Bsx_3483.2	0.938386	0.952312	0.938116
Cart1_0997.1	0.963675	0.965653	0.963746
Cart1_1275.1	0.970643	0.97127	0.970621
Cdx1_2245.1	0.948336	0.955471	0.948396
Cdx2_4272.1	0.948368	0.956109	0.948645
Cphx_3484.1	0.924809	0.937898	0.924875
Crx_3485.1	0.970795	0.974656	0.970096
Cutl1_3494.1	0.990339	0.991638	0.990277
Cutl1_3494.2	0.994035	0.993576	0.994107
Dbx1_3486.1	0.998208	0.99785	0.998208
Dbx2_3487.1	0.983013	0.981963	0.983052
Dlx1_1741.2	0.984428	0.984537	0.984429
Dlx2_2273.2	0.969877	0.970326	0.969887
Dlx3_1030.1	0.959318	0.962652	0.959225
Dlx4_3488.2	0.96241	0.965686	0.96249
Dlx5_3419.2	0.975364	0.97296	0.975454
Dmbx1_2277.1	0.972264	0.97649	0.972549
Dobox4_3956.2	0.95488	0.950278	0.954698
Dobox5_3493.1	0.979258	0.978583	0.979246
Duxl_1286.2	0.982177	0.983821	0.982152
Emx2_3420.1	0.962205	0.961167	0.962181
En1_3123.2	0.979771	0.981139	0.979747
En2_0952.1	0.955625	0.955861	0.955614
Esx1_3124.2	0.970128	0.971671	0.970155
Evx1_3952.2	0.943478	0.953044	0.943566
Evx2_2645.3	0.951345	0.962787	0.951371
Gbx1_2883.2	0.956476	0.958714	0.956426
Gbx2_3110.1	0.951278	0.956889	0.951398
Gsc_2327.3	0.967833	0.973092	0.967818
Gsh2_3990.2	0.9589	0.963956	0.9586

Hdx_3845.3	0.954485	0.974997	0.953599
Hlx1_2350.1	0.978519	0.982453	0.978569
Hlxb9_3422.1	0.956543	0.957321	0.956646
Hmbox1_2674.1	0.979771	0.98378	0.979966
Hmx1_3423.1	0.972405	0.965322	0.972434
Hmx2_3424.3	0.965615	0.957804	0.965202
Hmx3_3490.2	0.972603	0.965567	0.972641
Homez_1063.2	0.689982	0.862953	0.689984
Hoxa1_3425.1	0.981754	0.983773	0.981984
Hoxa10_2318.1	0.960711	0.96854	0.96057
Hoxa11_2218.1	0.932967	0.946671	0.933294
Hoxa13_3126.1	0.94304	0.951481	0.943282
Hoxa2_3079.1	0.965415	0.971713	0.965313
Hoxa3_2783.2	0.972715	0.977204	0.972879
Hoxa4_3426.1	0.960086	0.966457	0.959908
Hoxa5_3415.1	0.952434	0.961788	0.952428
Hoxa6_1040.1	0.954284	0.962655	0.954163
Hoxa7_2668.2	0.954081	0.960794	0.954143
Hoxa7_3750.1	0.978036	0.981656	0.977954
Hoxa9_2622.2	0.962073	0.965967	0.962312
Hoxb13_3479.1	0.943908	0.947327	0.943663
Hoxb3_1720.2	0.947503	0.957592	0.947554
Hoxb4_2627.1	0.964949	0.972101	0.96488
Hoxb5_3122.2	0.953391	0.95945	0.953361
Hoxb6_3428.2	0.973827	0.981207	0.973818
Hoxb7_3953.1	0.952527	0.95976	0.952786
Hoxb8_3780.2	0.951095	0.953782	0.951222
Hoxb9_3413.1	0.953047	0.96022	0.952751
Hoxc10_2779.2	0.921757	0.936002	0.92145
Hoxc11_3718.2	0.928441	0.941494	0.928513
Hoxc12_3480.1	0.926575	0.944211	0.926423
Hoxc13_3127.1	0.949592	0.955843	0.949692
Hoxc4_3491.1	0.960934	0.968576	0.960966
Hoxc5_2630.2	0.972711	0.978574	0.972639
Hoxc6_3954.2	0.980418	0.989191	0.980288
Hoxc8_3429.2	0.959226	0.969136	0.959296
Hoxc9_2367.2	0.965411	0.971516	0.965171
Hoxd1_3448.1	0.955007	0.955999	0.954988
Hoxd10_2368.2	0.970945	0.972568	0.970867
Hoxd11_3873.1	0.930915	0.944677	0.930785
Hoxd12_3481.1	0.927309	0.942135	0.927218

Hoxd13_2356.1	0.950097	0.955521	0.949683
Hoxd3_1742.2	0.947089	0.955778	0.94727
Hoxd8_2644.1	0.948726	0.961747	0.948476
Ipf1_3815.1	0.957739	0.968848	0.957681
Irx2_0900.3	0.870213	0.917014	0.870913
Irx3_0920.1	0.850819	0.890682	0.851249
Irx3_2226.1	0.863058	0.898073	0.86231
Irx4_2242.3	0.889442	0.952166	0.889272
Irx5_2385.1	0.865126	0.903247	0.865572
Irx6_2623.2	0.861907	0.937741	0.860516
Isl2_3430.1	0.945838	0.964972	0.945756
Isx_3445.1	0.948252	0.949826	0.948434
Lbx2_3869.2	0.972809	0.975054	0.972833
Lhx1_2240.2	0.972107	0.974907	0.972146
Lhx2_0953.2	0.96186	0.965319	0.961839
Lhx3_3431.1	0.963848	0.970589	0.963792
Lhx4_1719.2	0.951166	0.954197	0.95102
Lhx5_2279.1	0.9632	0.967859	0.9632
Lhx6_2272.1	0.949151	0.953276	0.949158
Lhx6_3432.1	0.948225	0.951551	0.948307
Lhx8_2247.2	0.95106	0.952191	0.950865
Lhx9_3492.1	0.967578	0.968812	0.967595
Lmx1a_2238.2	0.982678	0.985668	0.98263
Lmx1b_3433.2	0.982531	0.987954	0.98255
Meis1_2335.1	0.972558	0.982593	0.972701
Meox1_2310.2	0.972043	0.969738	0.972231
Mrg1_2246.2	0.968064	0.981803	0.967606
Mrg2_2302.1	0.975857	0.987041	0.97566
Msx1_3031.2	0.950289	0.955246	0.95001
Msx2_3449.1	0.980224	0.9811	0.980177
Msx3_3206.1	0.986552	0.984249	0.986465
Nkx1-1_3856.3	0.934221	0.946638	0.933868
Nkx1-2_3214.1	0.959507	0.964076	0.959742
Nkx2-2_2823.1	0.969605	0.972323	0.96966
Nkx2-3_3435.1	0.963443	0.970735	0.963454
Nkx2-4_3074.1	0.971911	0.972365	0.972031
Nkx2-5_3436.1	0.978092	0.981117	0.978296
Nkx2-6_3437.1	0.97248	0.977037	0.972326
Nkx2-9_3082.1	0.944965	0.950491	0.944346
Nkx3-1_2923.2	0.9813	0.986022	0.981424
Nkx6-1_2825.1	0.969219	0.968319	0.969319

Nkx6-1_2825.2	0.96151	0.960122	0.961317
Nkx6-3_3446.1	0.968697	0.971031	0.968429
Obox1_3970.2	0.983788	0.982771	0.983723
Obox2_3438.2	0.940575	0.953573	0.939715
Obox3_3439.1	0.958956	0.965636	0.958882
Obox5_2284.1	0.955578	0.964393	0.955746
Obox5_3963.2	0.954287	0.965066	0.954183
Obox6_3440.2	0.982077	0.985541	0.982115
Og2x_3719.1	0.981773	0.985086	0.981719
Otp_3496.1	0.962344	0.964254	0.962291
Otx1_2325.1	0.968277	0.970359	0.968413
Otx2_3441.1	0.97175	0.973207	0.971973
Pax4_3989.2	0.955742	0.96674	0.955814
Pax6_3838.3	0.980793	0.986445	0.980471
Pax7_3783.1	0.953363	0.96334	0.953306
Pbx1_3203.1	0.975158	0.977373	0.974981
Phox2a_3947.1	0.957815	0.962942	0.957762
Phox2b_3948.1	0.950782	0.955529	0.950844
Pitx1_2312.1	0.987292	0.985422	0.987253
Pitx2_2274.3	0.984951	0.983274	0.98481
Pitx3_3497.2	0.986016	0.984998	0.986043
Pknox1_2364.2	0.967268	0.981762	0.967324
Pknox2_3077.2	0.978894	0.987409	0.979142
Pou1f1_3818.1	0.936644	0.944854	0.936426
Pou2f1_3081.2	0.935361	0.942553	0.935246
Pou2f2_3748.1	0.93892	0.936155	0.938917
Pou2f3_3986.2	0.927242	0.927048	0.927308
Pou3f1_3819.1	0.933941	0.945397	0.933874
Pou3f2_2824.1	0.952801	0.956697	0.952892
Pou3f3_3235.2	0.912864	0.925416	0.91254
Pou3f4_3773.1	0.938219	0.945985	0.937996
Pou4f3_2791.1	0.952621	0.959574	0.952668
Pou6f1_1731.2	0.97104	0.972902	0.970918
Pou6f1_3733.1	0.968923	0.973187	0.96862
Prop1_3949.1	0.981009	0.984319	0.981051
Prrx1_3442.1	0.956296	0.962402	0.956457
Prrx2_3072.1	0.950342	0.949753	0.950244
Rax_3443.1	0.960608	0.963481	0.960551
Rhox11_1765.2	0.892358	0.915444	0.892637
Rhox11_2205.1	0.907094	0.927628	0.907127
Rhox6_4251.1	0.968882	0.961822	0.968812

Shox2_2641.2	0.969907	0.96668	0.969791
Six1_0935.2	0.881832	0.93761	0.881813
Six2_2307.2	0.865967	0.920068	0.866337
Six3_1732.2	0.904264	0.940388	0.904201
Six4_2860.1	0.903227	0.912851	0.903222
Six6_2267.4	0.908622	0.949128	0.908502
Six6_2267.5	0.896807	0.933403	0.896799
Tcf1_2666.2	0.91938	0.941355	0.91951
Tcf2_0913.2	0.958978	0.970274	0.958893
Tgif1_2342.2	0.965543	0.977833	0.965347
Tgif2_3451.1	0.977969	0.986237	0.977862
Titf1_1722.2	0.961747	0.968131	0.961479
Tlx2_3498.2	0.987431	0.987921	0.987132
Uncx4.1_2281.2	0.950894	0.956832	0.951077
Vax1_3499.1	0.955485	0.963658	0.95559
Vax2_3500.1	0.958388	0.963938	0.958629
Vsx1_1728.1	0.955676	0.953503	0.955677

Supplementary Table S7:

MLR coefficients for the DNA sequence and shape-augmented models, using mouse PBM data (14).
See Excel spreadsheet in separate Supplementary Data file for data.

Supplementary Table S8:

DNA binding site logos representing PWMs derived from 8-mers that were predicted by the DNA sequence and shape-augmented models to be bound to the protein, using mouse PBM data (14). Since some of the homeodomains did not have any 8-mers with predicted E-scores > 0.45, we used the 8-mers with the top 5% E-scores for generating the binding site logos that we included.

Column 1: Logos derived from bound 8−mers derived from universal PBM.
Column 2: Logos derived from bound 8−mers predicted by the DNA sequence model.
Column 3: Logos derived from bound 8−mers predicted by the DNA shape-augmented model.
Column 4: Logos derived from bound 8−mers predicted by a model that combines DNA sequence with shuffled shape parameters.

See following pages for data.

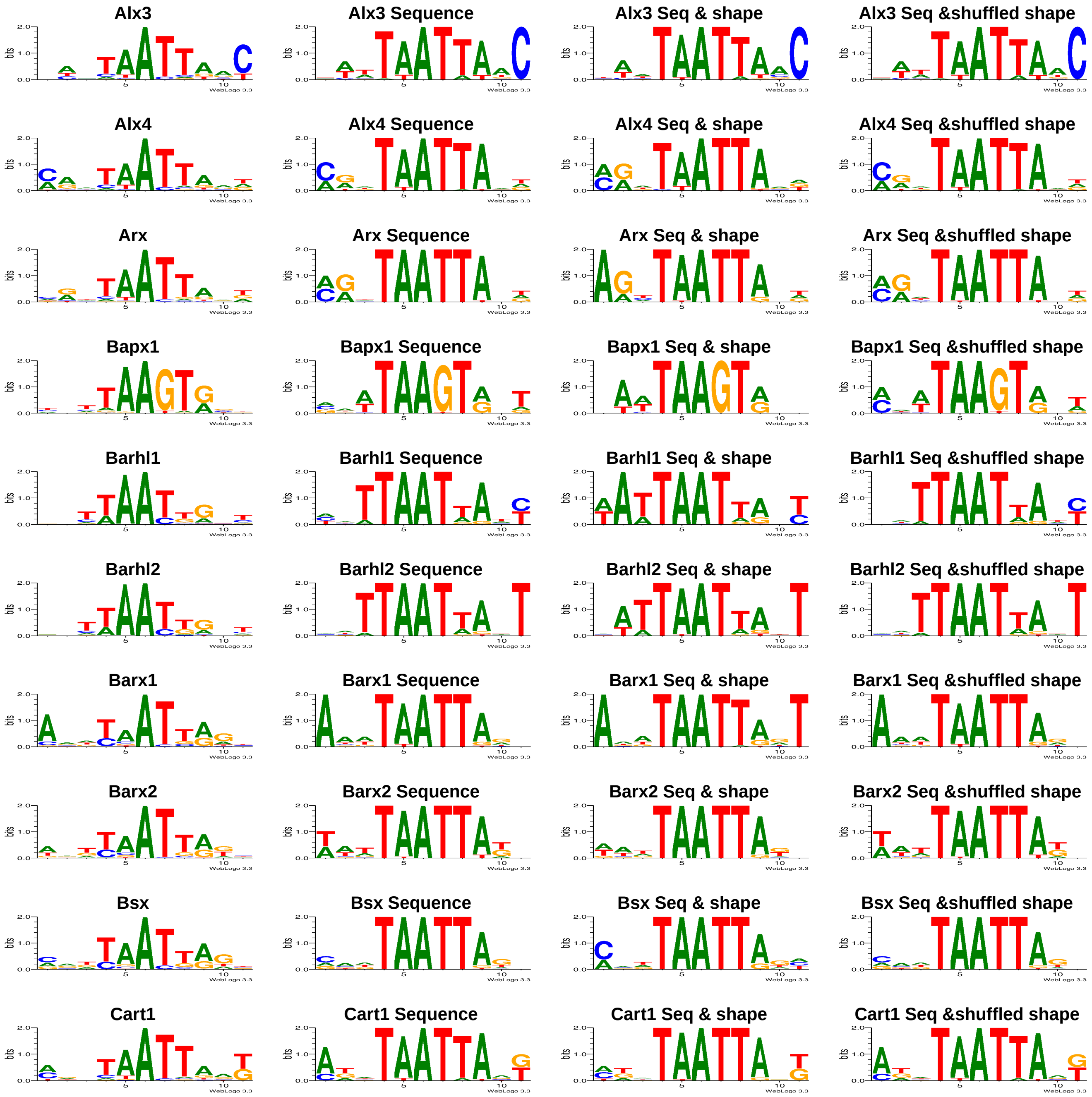
Alx3
Alx3 Sequence
Alx3 Seq & shape
Alx3 Seq &shuffled shape
Alx4
Alx4 Sequence
Alx4 Seq & shape
Alx4 Seq &shuffled shape
Arx
Arx Sequence
Arx Seq & shape
Arx Seq &shuffled shape
Bapx1
Bapx1 Sequence
Bapx1 Seq & shape
Bapx1 Seq &shuffled shape
Barhl1
Barhl1 Sequence
Barhl1 Seq & shape
Barhl1 Seq &shuffled shape
Barhl2
Barhl2 Sequence
Barhl2 Seq & shape
Barhl2 Seq &shuffled shape
Barx1
Barx1 Sequence
Barx1 Seq & shape
Barx1 Seq &shuffled shape
Barx2
Barx2 Sequence
Barx2 Seq & shape
Barx2 Seq &shuffled shape
Bsx
Bsx Sequence
Bsx Seq & shape
Bsx Seq &shuffled shape
Cart1
Cart1 Sequence
Cart1 Seq & shape
Cart1 Seq &shuffled shape

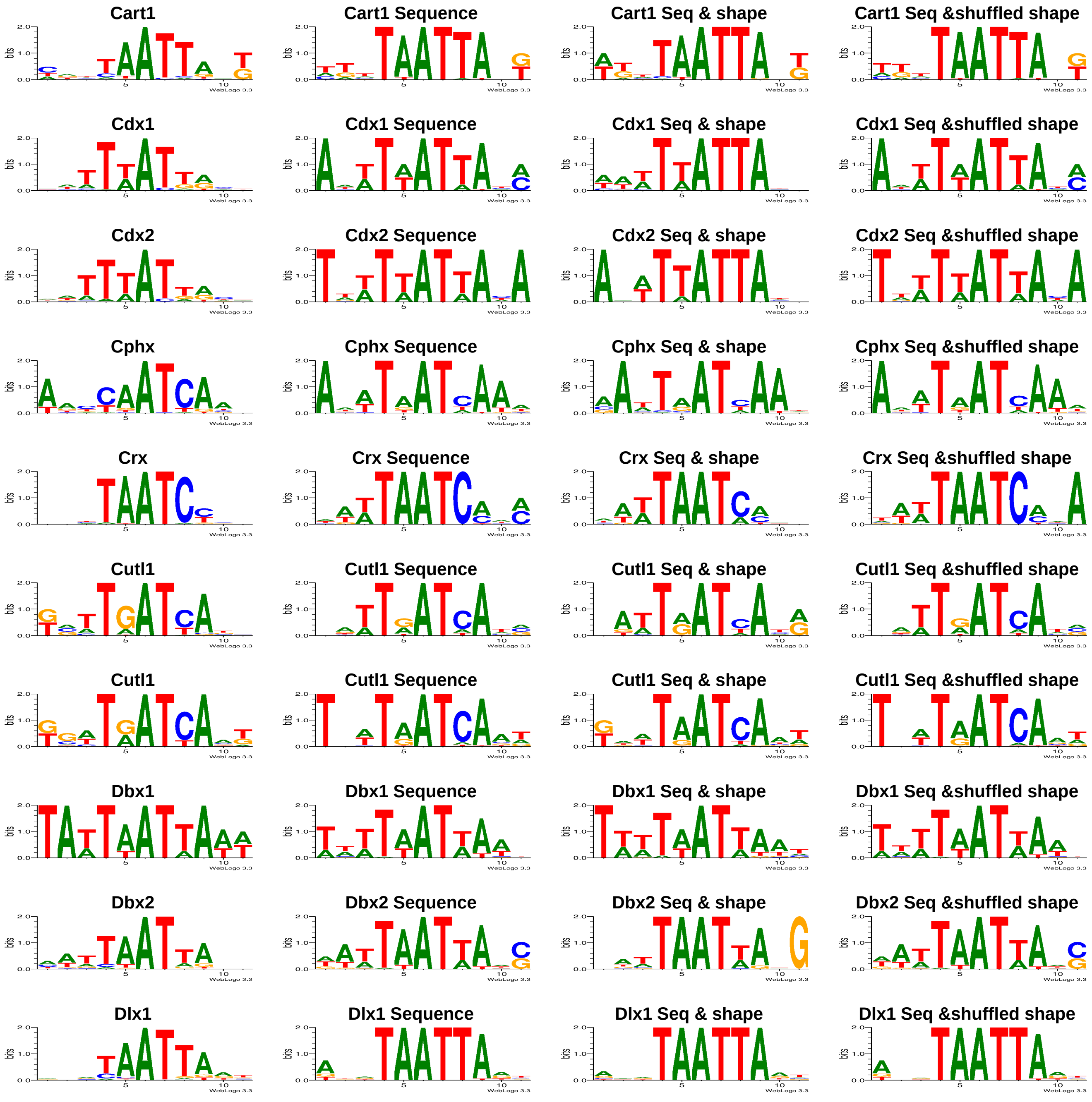
Cart1
Cart1 Sequence
Cart1 Seq & shape
Cart1 Seq &shuffled shape
Cdx1
Cdx1 Sequence
Cdx1 Seq & shape
Cdx1 Seq &shuffled shape
Cdx2
Cdx2 Sequence
Cdx2 Seq & shape
Cdx2 Seq &shuffled shape
Cphx
Cphx Sequence
Cphx Seq & shape
Cphx Seq &shuffled shape
Crx
Crx Sequence
Crx Seq & shape
Crx Seq &shuffled shape
Cutl1
Cutl1 Sequence
Cutl1 Seq & shape
Cutl1 Seq &shuffled shape
Cutl1
Cutl1 Sequence
Cutl1 Seq & shape
Cutl1 Seq &shuffled shape
Dbx1
Dbx1 Sequence
Dbx1 Seq & shape
Dbx1 Seq &shuffled shape
Dbx2
Dbx2 Sequence
Dbx2 Seq & shape
Dbx2 Seq &shuffled shape
Dlx1
Dlx1 Sequence
Dlx1 Seq & shape
Dlx1 Seq &shuffled shape

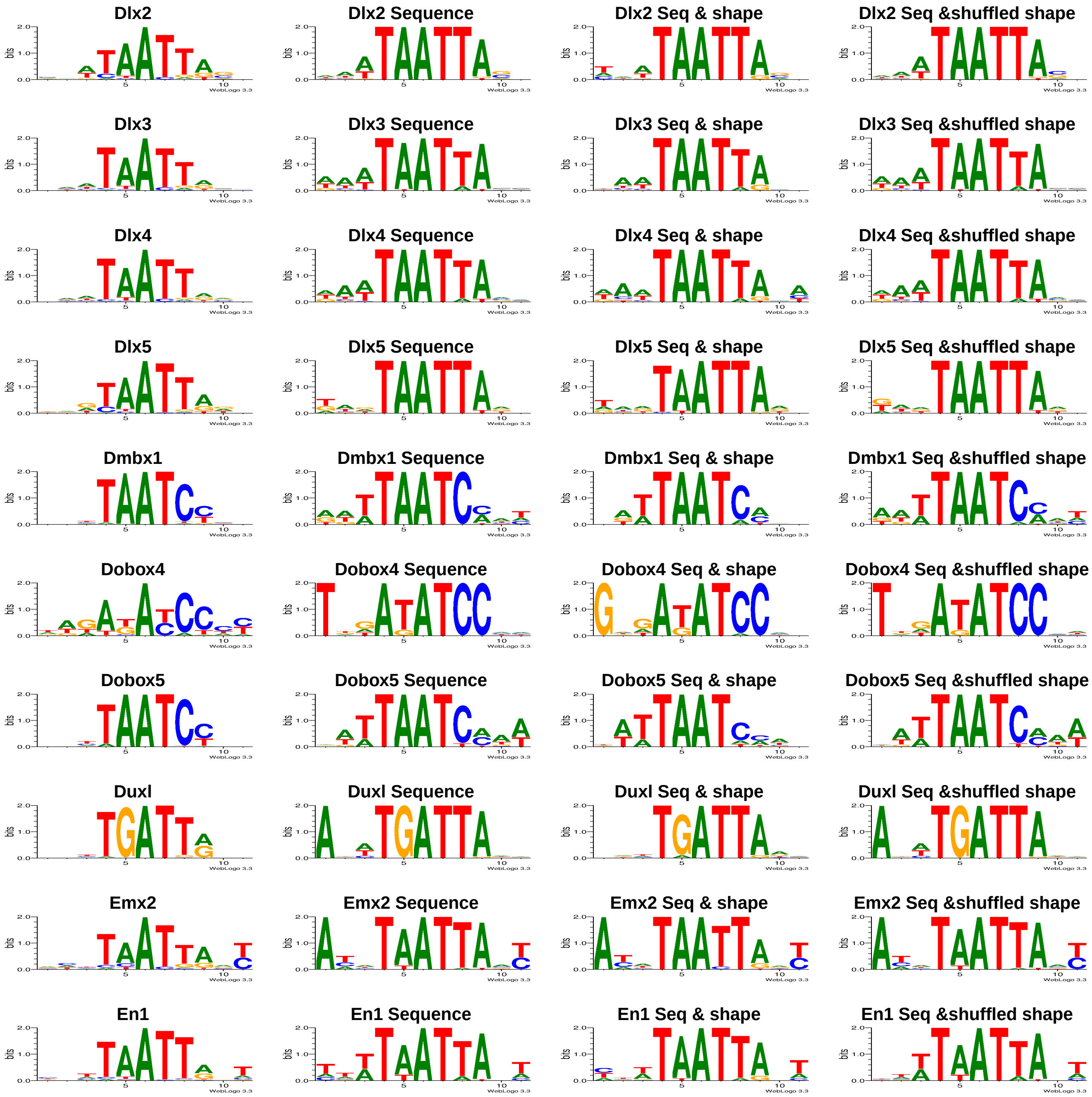
Dlx2
Dlx2 Sequence
Dlx2 Seq & shape
Dlx2 Seq &shuffled shape
Dlx3
Dlx3 Sequence
Dlx3 Seq & shape
Dlx3 Seq &shuffled shape
Dlx4
Dlx4 Sequence
Dlx4 Seq & shape
Dlx4 Seq &shuffled shape
Dlx5
Dlx5 Sequence
Dlx5 Seq & shape
Dlx5 Seq &shuffled shape
Dmbx1
Dmbx1 Sequence
Dmbx1 Seq & shape
Dmbx1 Seq &shuffled shape
Dobox4
Dobox4 Sequence
Dobox4 Seq & shape
Dobox4 Seq &shuffled shape
Dobox5
Dobox5 Sequence
Dobox5 Seq & shape
Dobox5 Seq &shuffled shape
Duxl
Duxl Sequence
Duxl Seq & shape
Duxl Seq &shuffled shape
Emx2
Emx2 Sequence
Emx2 Seq & shape
Emx2 Seq &shuffled shape
En1
En1 Sequence
En1 Seq & shape
En1 Seq &shuffled shape

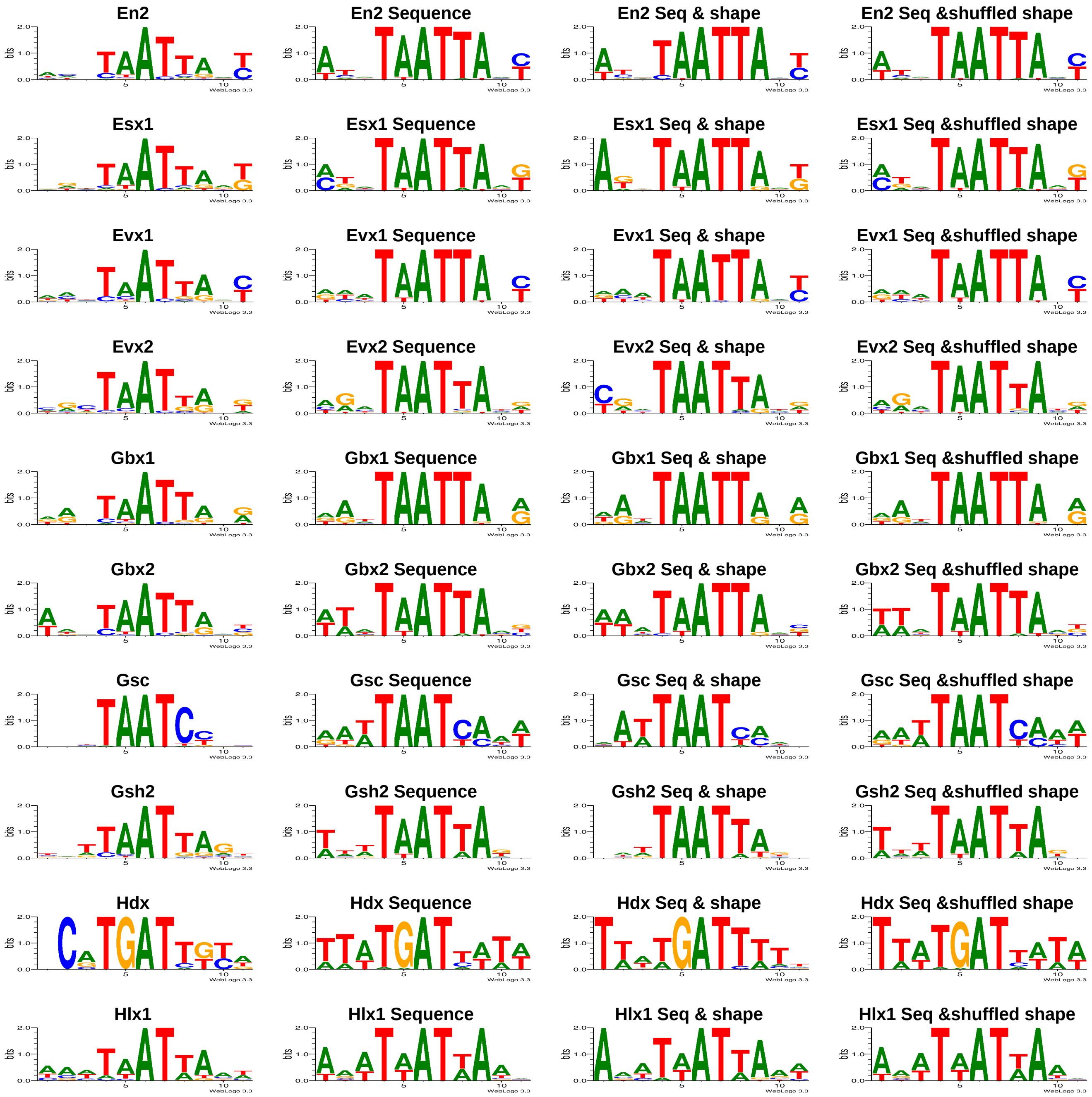
En2
En2 Sequence
En2 Seq & shape
En2 Seq &shuffled shape
Esx1
Esx1 Sequence
Esx1 Seq & shape
Esx1 Seq &shuffled shape
Evx1
Evx1 Sequence
Evx1 Seq & shape
Evx1 Seq &shuffled shape
Evx2
Evx2 Sequence
Evx2 Seq & shape
Evx2 Seq &shuffled shape
Gbx1
Gbx1 Sequence
Gbx1 Seq & shape
Gbx1 Seq &shuffled shape
Gbx2
Gbx2 Sequence
Gbx2 Seq & shape
Gbx2 Seq &shuffled shape
Gsc
Gsc Sequence
Gsc Seq & shape
Gsc Seq &shuffled shape
Gsh2
Gsh2 Sequence
Gsh2 Seq & shape
Gsh2 Seq &shuffled shape
Hdx
Hdx Sequence
Hdx Seq & shape
Hdx Seq &shuffled shape
Hlx1
Hlx1 Sequence
Hlx1 Seq & shape
Hlx1 Seq &shuffled shape
bits
WebLogo 3.3

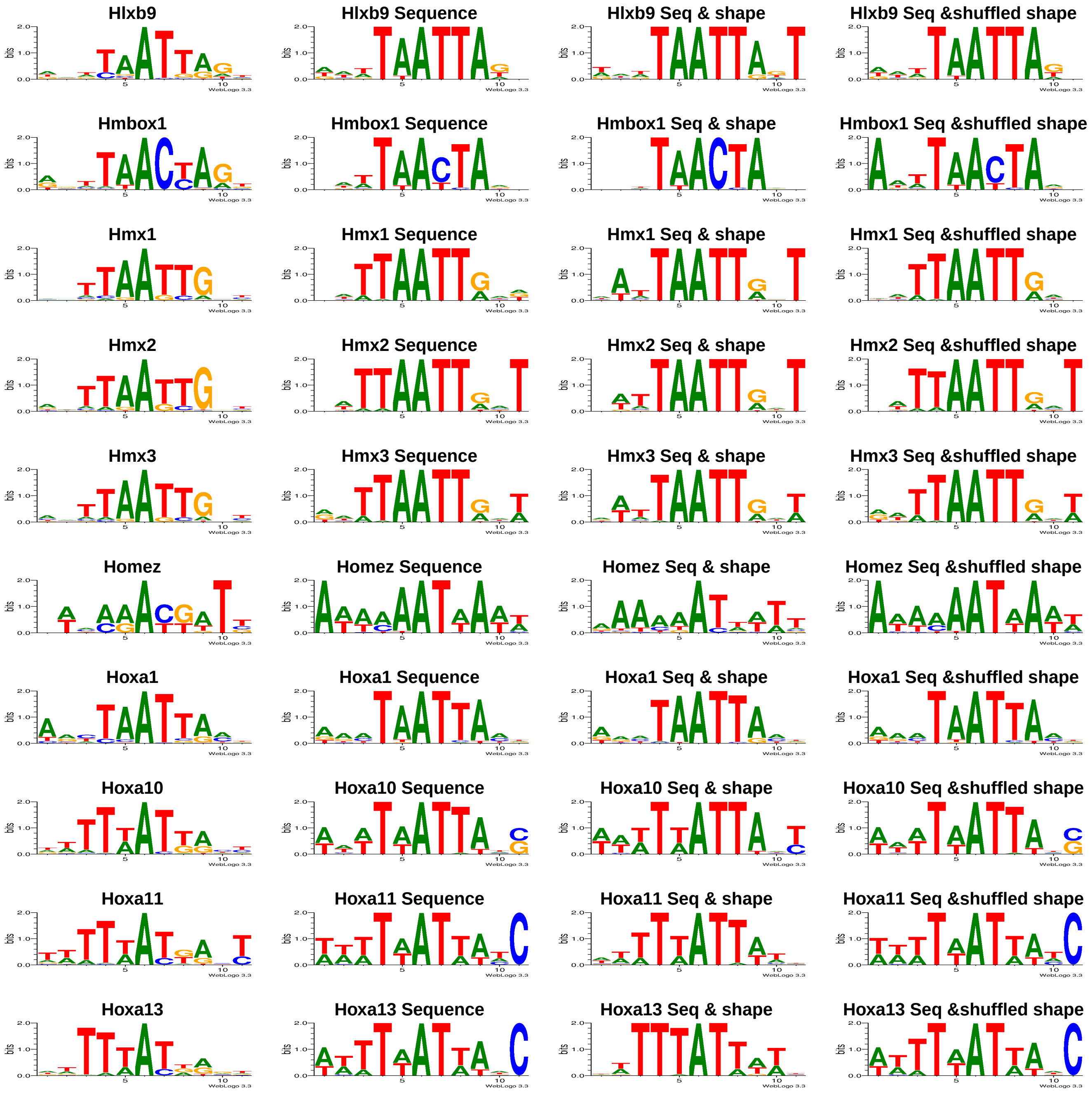
Hlxb9
Hlxb9 Sequence
Hlxb9 Seq & shape
Hlxb9 Seq &shuffled shape
Hmbox1
Hmbox1 Sequence
Hmbox1 Seq & shape
Hmbox1 Seq &shuffled shape
Hmx1
Hmx1 Sequence
Hmx1 Seq & shape
Hmx1 Seq &shuffled shape
Hmx2
Hmx2 Sequence
Hmx2 Seq & shape
Hmx2 Seq &shuffled shape
Hmx3
Hmx3 Sequence
Hmx3 Seq & shape
Hmx3 Seq &shuffled shape
Homez
Homez Sequence
Homez Seq & shape
Homez Seq &shuffled shape
Hoxa1
Hoxa1 Sequence
Hoxa1 Seq & shape
Hoxa1 Seq &shuffled shape
Hoxa10
Hoxa10 Sequence
Hoxa10 Seq & shape
Hoxa10 Seq &shuffled shape
Hoxa11
Hoxa11 Sequence
Hoxa11 Seq & shape
Hoxa11 Seq &shuffled shape
Hoxa13
Hoxa13 Sequence
Hoxa13 Seq & shape
Hoxa13 Seq &shuffled shape

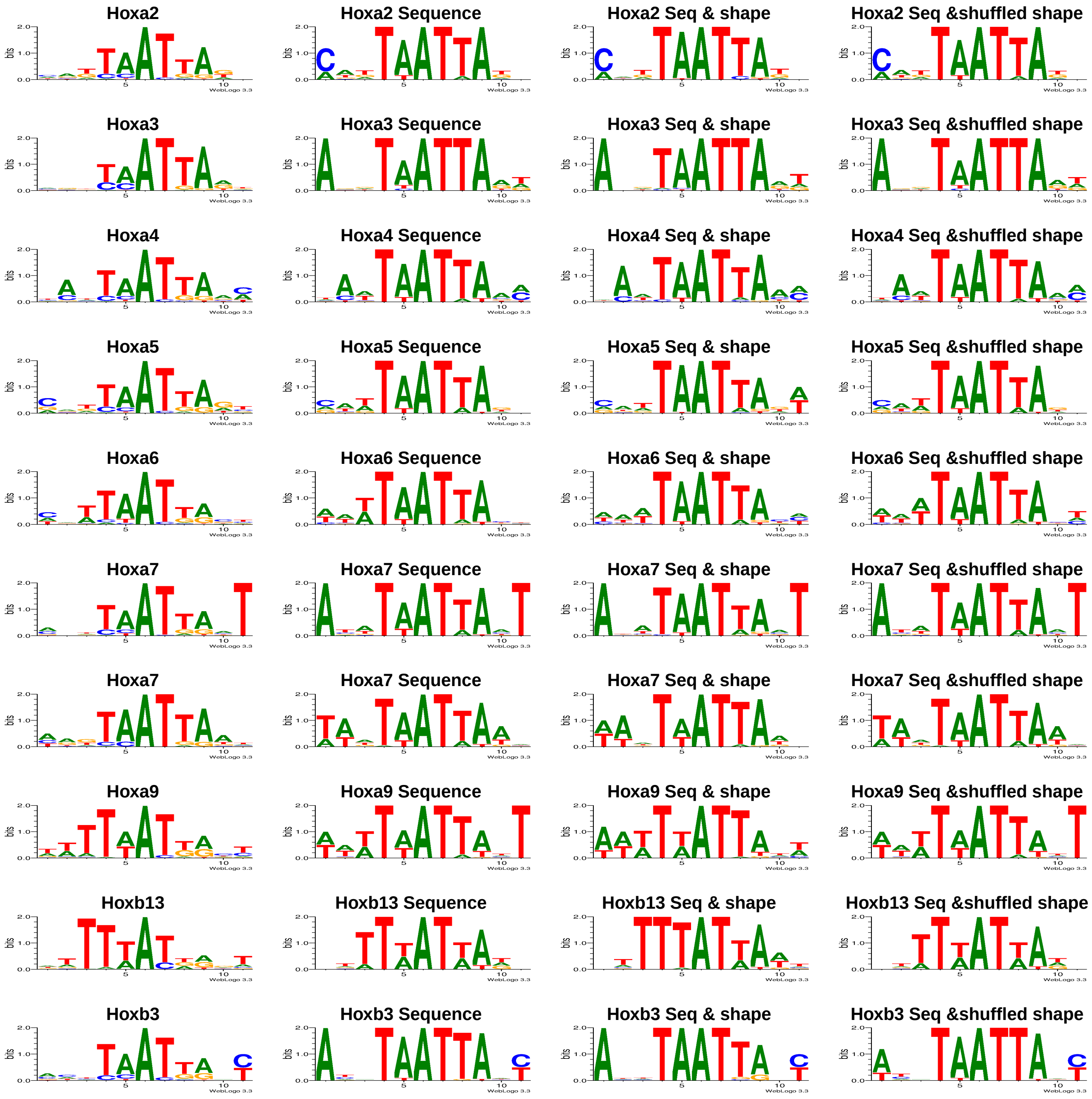
Hoxa2
Hoxa2 Sequence
Hoxa2 Seq & shape
Hoxa2 Seq &shuffled shape
Hoxa3
Hoxa3 Sequence
Hoxa3 Seq & shape
Hoxa3 Seq &shuffled shape
Hoxa4
Hoxa4 Sequence
Hoxa4 Seq & shape
Hoxa4 Seq &shuffled shape
Hoxa5
Hoxa5 Sequence
Hoxa5 Seq & shape
Hoxa5 Seq &shuffled shape
Hoxa6
Hoxa6 Sequence
Hoxa6 Seq & shape
Hoxa6 Seq &shuffled shape
Hoxa7
Hoxa7 Sequence
Hoxa7 Seq & shape
Hoxa7 Seq &shuffled shape
Hoxa7
Hoxa7 Sequence
Hoxa7 Seq & shape
Hoxa7 Seq &shuffled shape
Hoxa9
Hoxa9 Sequence
Hoxa9 Seq & shape
Hoxa9 Seq &shuffled shape
Hoxb13
Hoxb13 Sequence
Hoxb13 Seq & shape
Hoxb13 Seq &shuffled shape
Hoxb3
Hoxb3 Sequence
Hoxb3 Seq & shape
Hoxb3 Seq &shuffled shape

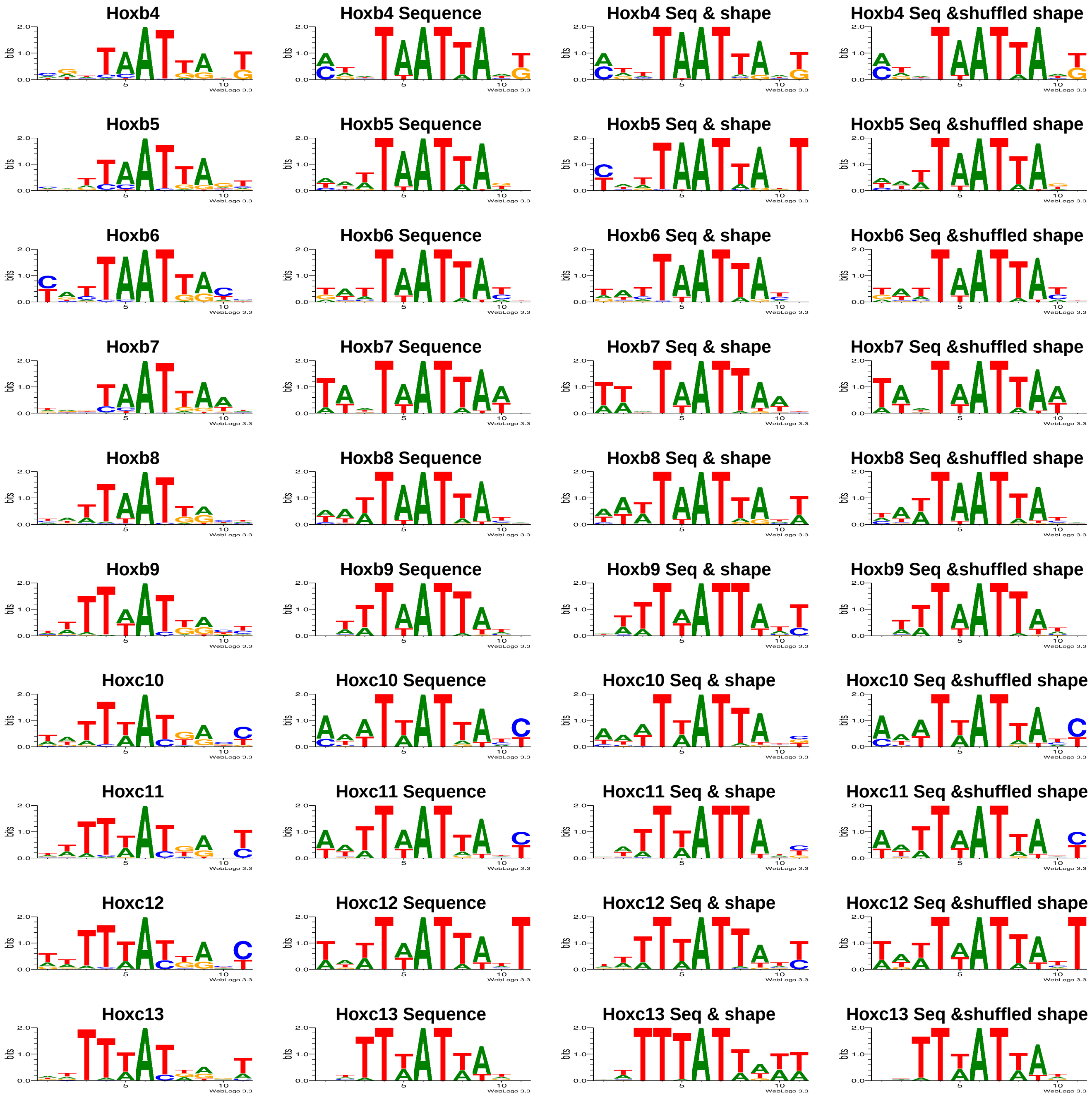
Hoxb4
Hoxb4 Sequence
Hoxb4 Seq & shape
Hoxb4 Seq &shuffled shape
Hoxb5
Hoxb5 Sequence
Hoxb5 Seq & shape
Hoxb5 Seq &shuffled shape
Hoxb6
Hoxb6 Sequence
Hoxb6 Seq & shape
Hoxb6 Seq &shuffled shape
Hoxb7
Hoxb7 Sequence
Hoxb7 Seq & shape
Hoxb7 Seq &shuffled shape
Hoxb8
Hoxb8 Sequence
Hoxb8 Seq & shape
Hoxb8 Seq &shuffled shape
Hoxb9
Hoxb9 Sequence
Hoxb9 Seq & shape
Hoxb9 Seq &shuffled shape
Hoxc10
Hoxc10 Sequence
Hoxc10 Seq & shape
Hoxc10 Seq &shuffled shape
Hoxc11
Hoxc11 Sequence
Hoxc11 Seq & shape
Hoxc11 Seq &shuffled shape
Hoxc12
Hoxc12 Sequence
Hoxc12 Seq & shape
Hoxc12 Seq &shuffled shape
Hoxc13
Hoxc13 Sequence
Hoxc13 Seq & shape
Hoxc13 Seq &shuffled shape

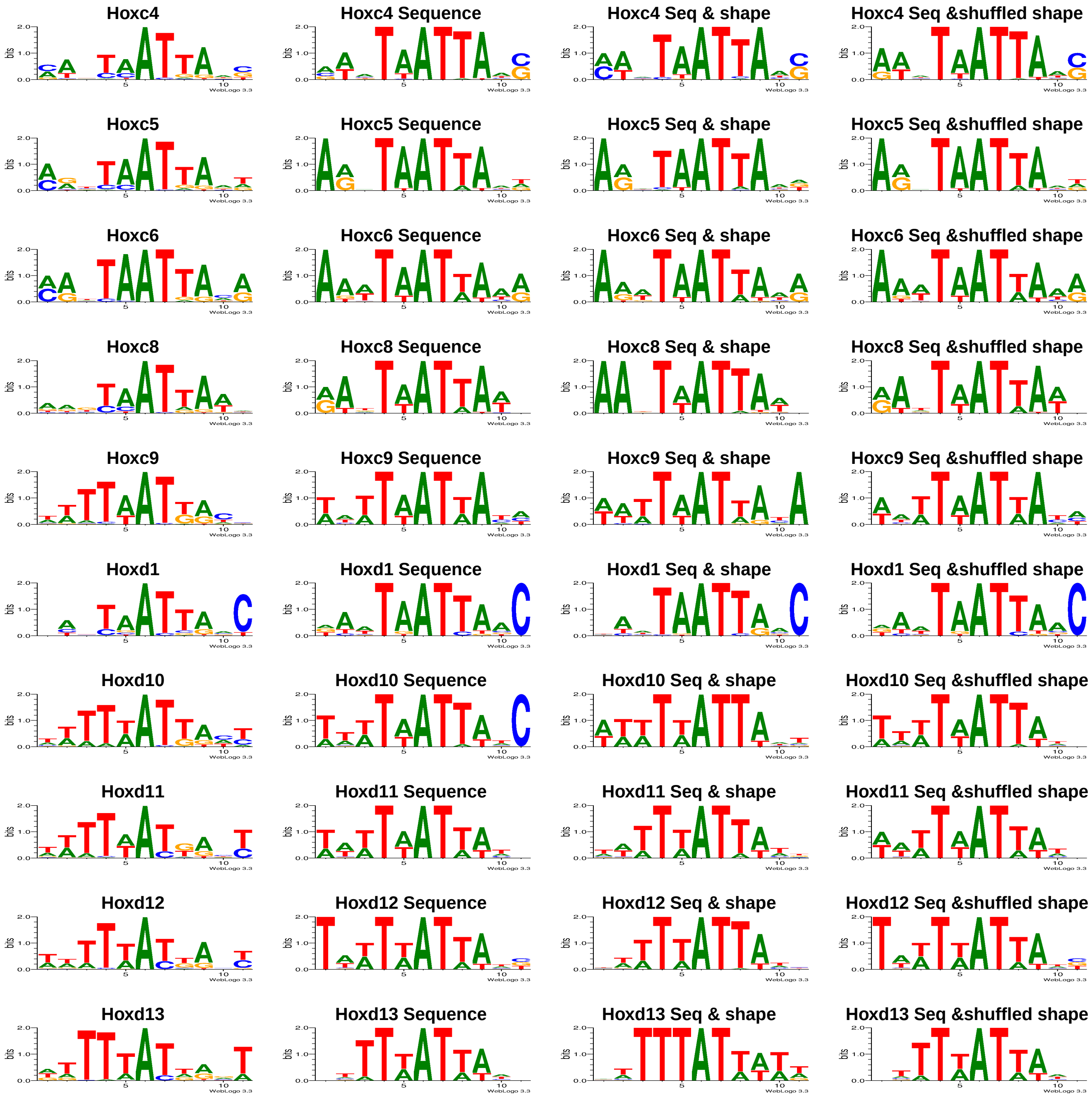
Hoxc4
Hoxc4 Sequence
Hoxc4 Seq & shape
Hoxc4 Seq &shuffled shape
Hoxc5
Hoxc5 Sequence
Hoxc5 Seq & shape
Hoxc5 Seq &shuffled shape
Hoxc6
Hoxc6 Sequence
Hoxc6 Seq & shape
Hoxc6 Seq &shuffled shape
Hoxc8
Hoxc8 Sequence
Hoxc8 Seq & shape
Hoxc8 Seq &shuffled shape
Hoxc9
Hoxc9 Sequence
Hoxc9 Seq & shape
Hoxc9 Seq &shuffled shape
Hoxd1
Hoxd1 Sequence
Hoxd1 Seq & shape
Hoxd1 Seq &shuffled shape
Hoxd10
Hoxd10 Sequence
Hoxd10 Seq & shape
Hoxd10 Seq &shuffled shape
Hoxd11
Hoxd11 Sequence
Hoxd11 Seq & shape
Hoxd11 Seq &shuffled shape
Hoxd12
Hoxd12 Sequence
Hoxd12 Seq & shape
Hoxd12 Seq &shuffled shape
Hoxd13
Hoxd13 Sequence
Hoxd13 Seq & shape
Hoxd13 Seq &shuffled shape

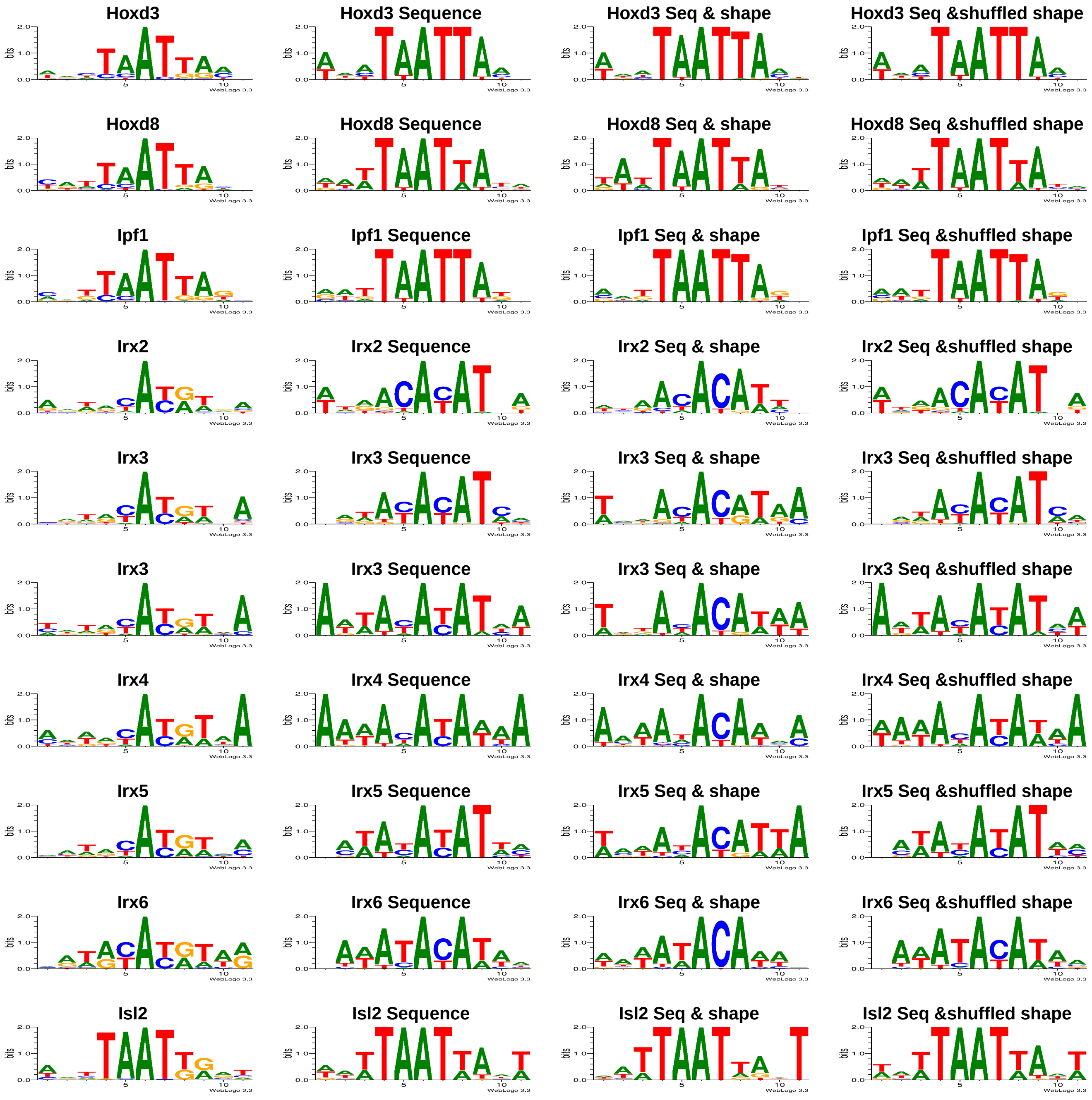
Hoxd3
Hoxd3 Sequence
Hoxd3 Seq & shape
Hoxd3 Seq &shuffled shape
Hoxd8
Hoxd8 Sequence
Hoxd8 Seq & shape
Hoxd8 Seq &shuffled shape
Ipf1
Ipf1 Sequence
Ipf1 Seq & shape
Ipf1 Seq &shuffled shape
Irx2
Irx2 Sequence
Irx2 Seq & shape
Irx2 Seq &shuffled shape
Irx3
Irx3 Sequence
Irx3 Seq & shape
Irx3 Seq &shuffled shape
Irx3
Irx3 Sequence
Irx3 Seq & shape
Irx3 Seq &shuffled shape
Irx4
Irx4 Sequence
Irx4 Seq & shape
Irx4 Seq &shuffled shape
Irx5
Irx5 Sequence
Irx5 Seq & shape
Irx5 Seq &shuffled shape
Irx6
Irx6 Sequence
Irx6 Seq & shape
Irx6 Seq &shuffled shape
Isl2
Isl2 Sequence
Isl2 Seq & shape
Isl2 Seq &shuffled shape

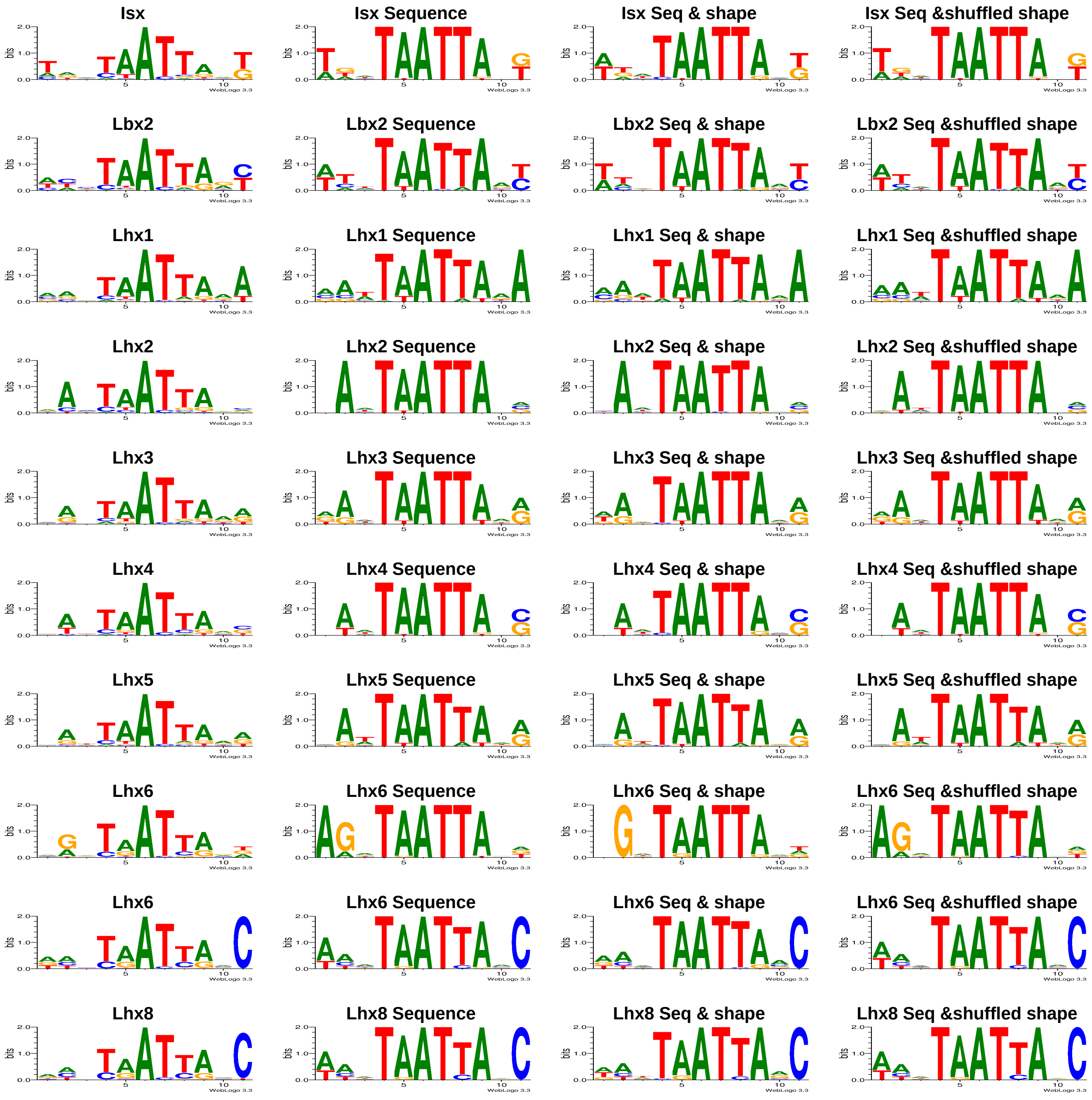
Isx
Isx Sequence
Isx Seq & shape
Isx Seq &shuffled shape
Lbx2
Lbx2 Sequence
Lbx2 Seq & shape
Lbx2 Seq &shuffled shape
Lhx1
Lhx1 Sequence
Lhx1 Seq & shape
Lhx1 Seq &shuffled shape
Lhx2
Lhx2 Sequence
Lhx2 Seq & shape
Lhx2 Seq &shuffled shape
Lhx3
Lhx3 Sequence
Lhx3 Seq & shape
Lhx3 Seq &shuffled shape
Lhx4
Lhx4 Sequence
Lhx4 Seq & shape
Lhx4 Seq &shuffled shape
Lhx5
Lhx5 Sequence
Lhx5 Seq & shape
Lhx5 Seq &shuffled shape
Lhx6
Lhx6 Sequence
Lhx6 Seq & shape
Lhx6 Seq &shuffled shape
Lhx6
Lhx6 Sequence
Lhx6 Seq & shape
Lhx6 Seq &shuffled shape
Lhx8
Lhx8 Sequence
Lhx8 Seq & shape
Lhx8 Seq &shuffled shape

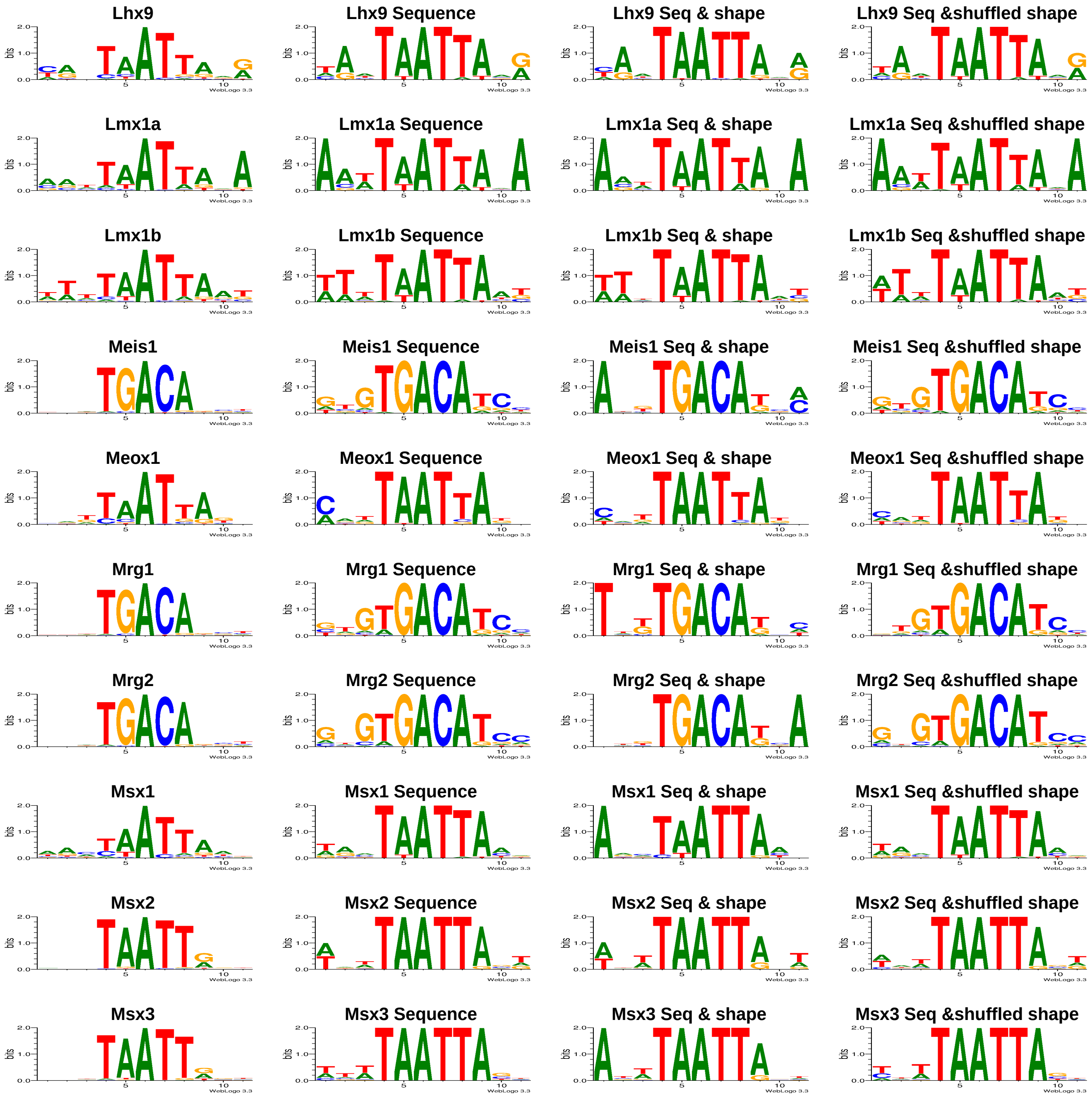
Lhx9
Lhx9 Sequence
Lhx9 Seq & shape
Lhx9 Seq &shuffled shape
Lmx1a
Lmx1a Sequence
Lmx1a Seq & shape
Lmx1a Seq &shuffled shape
Lmx1b
Lmx1b Sequence
Lmx1b Seq & shape
Lmx1b Seq &shuffled shape
Meis1
Meis1 Sequence
Meis1 Seq & shape
Meis1 Seq &shuffled shape
Meox1
Meox1 Sequence
Meox1 Seq & shape
Meox1 Seq &shuffled shape
Mrg1
Mrg1 Sequence
Mrg1 Seq & shape
Mrg1 Seq &shuffled shape
Mrg2
Mrg2 Sequence
Mrg2 Seq & shape
Mrg2 Seq &shuffled shape
Msx1
Msx1 Sequence
Msx1 Seq & shape
Msx1 Seq &shuffled shape
Msx2
Msx2 Sequence
Msx2 Seq & shape
Msx2 Seq &shuffled shape
Msx3
Msx3 Sequence
Msx3 Seq & shape
Msx3 Seq &shuffled shape

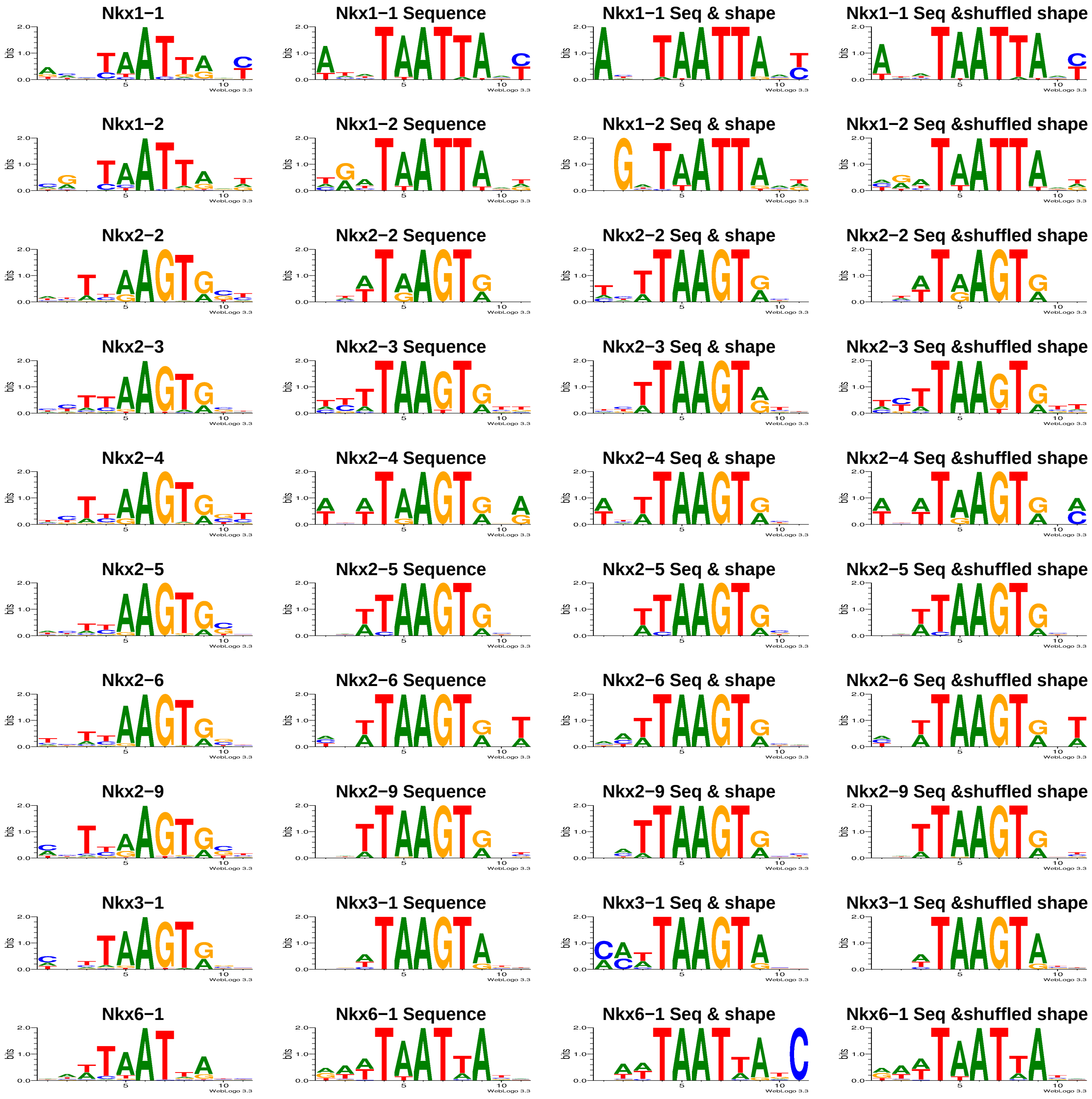

Nkx1−1
Nkx1−1 Sequence
Nkx1−1 Seq & shape
Nkx1−1 Seq &shuffled shape
Nkx1−2
Nkx1−2 Sequence
Nkx1−2 Seq & shape
Nkx1−2 Seq &shuffled shape
Nkx2−2
Nkx2−2 Sequence
Nkx2−2 Seq & shape
Nkx2−2 Seq &shuffled shape
Nkx2−3
Nkx2−3 Sequence
Nkx2−3 Seq & shape
Nkx2−3 Seq &shuffled shape
Nkx2−4
Nkx2−4 Sequence
Nkx2−4 Seq & shape
Nkx2−4 Seq &shuffled shape
Nkx2−5
Nkx2−5 Sequence
Nkx2−5 Seq & shape
Nkx2−5 Seq &shuffled shape
Nkx2−6
Nkx2−6 Sequence
Nkx2−6 Seq & shape
Nkx2−6 Seq &shuffled shape
Nkx2−9
Nkx2−9 Sequence
Nkx2−9 Seq & shape
Nkx2−9 Seq &shuffled shape
Nkx3−1
Nkx3−1 Sequence
Nkx3−1 Seq & shape
Nkx3−1 Seq &shuffled shape
Nkx6−1
Nkx6−1 Sequence
Nkx6−1 Seq & shape
Nkx6−1 Seq &shuffled shape

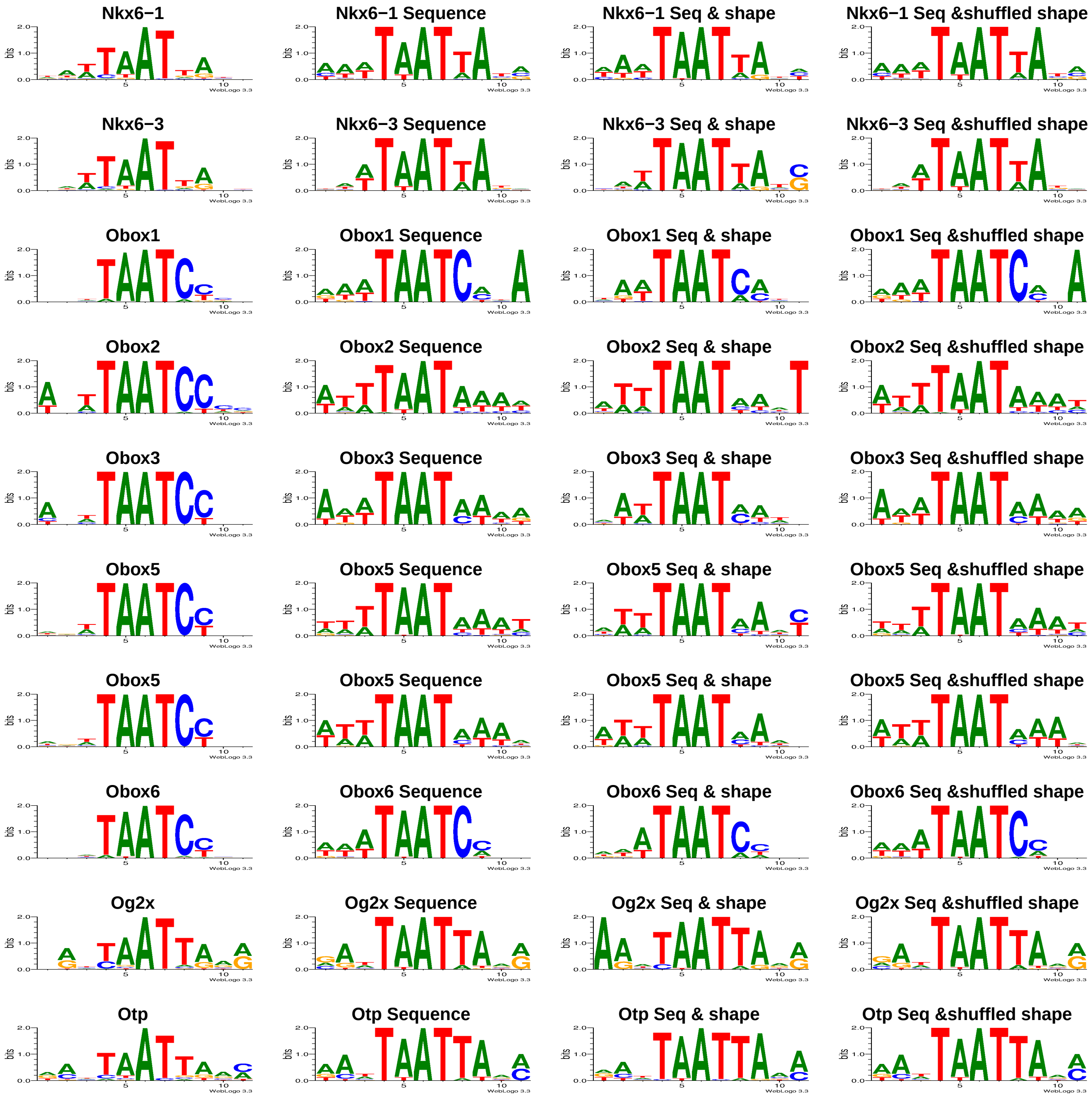
Nkx6−1
Nkx6−1 Sequence
Nkx6−1 Seq & shape
Nkx6−1 Seq &shuffled shape
Nkx6−3
Nkx6−3 Sequence
Nkx6−3 Seq & shape
Nkx6−3 Seq &shuffled shape
Obox1
Obox1 Sequence
Obox1 Seq & shape
Obox1 Seq &shuffled shape
Obox2
Obox2 Sequence
Obox2 Seq & shape
Obox2 Seq &shuffled shape
Obox3
Obox3 Sequence
Obox3 Seq & shape
Obox3 Seq &shuffled shape
Obox5
Obox5 Sequence
Obox5 Seq & shape
Obox5 Seq &shuffled shape
Obox5
Obox5 Sequence
Obox5 Seq & shape
Obox5 Seq &shuffled shape
Obox6
Obox6 Sequence
Obox6 Seq & shape
Obox6 Seq &shuffled shape
Og2x
Og2x Sequence
Og2x Seq & shape
Og2x Seq &shuffled shape
Otp
Otp Sequence
Otp Seq & shape
Otp Seq &shuffled shape

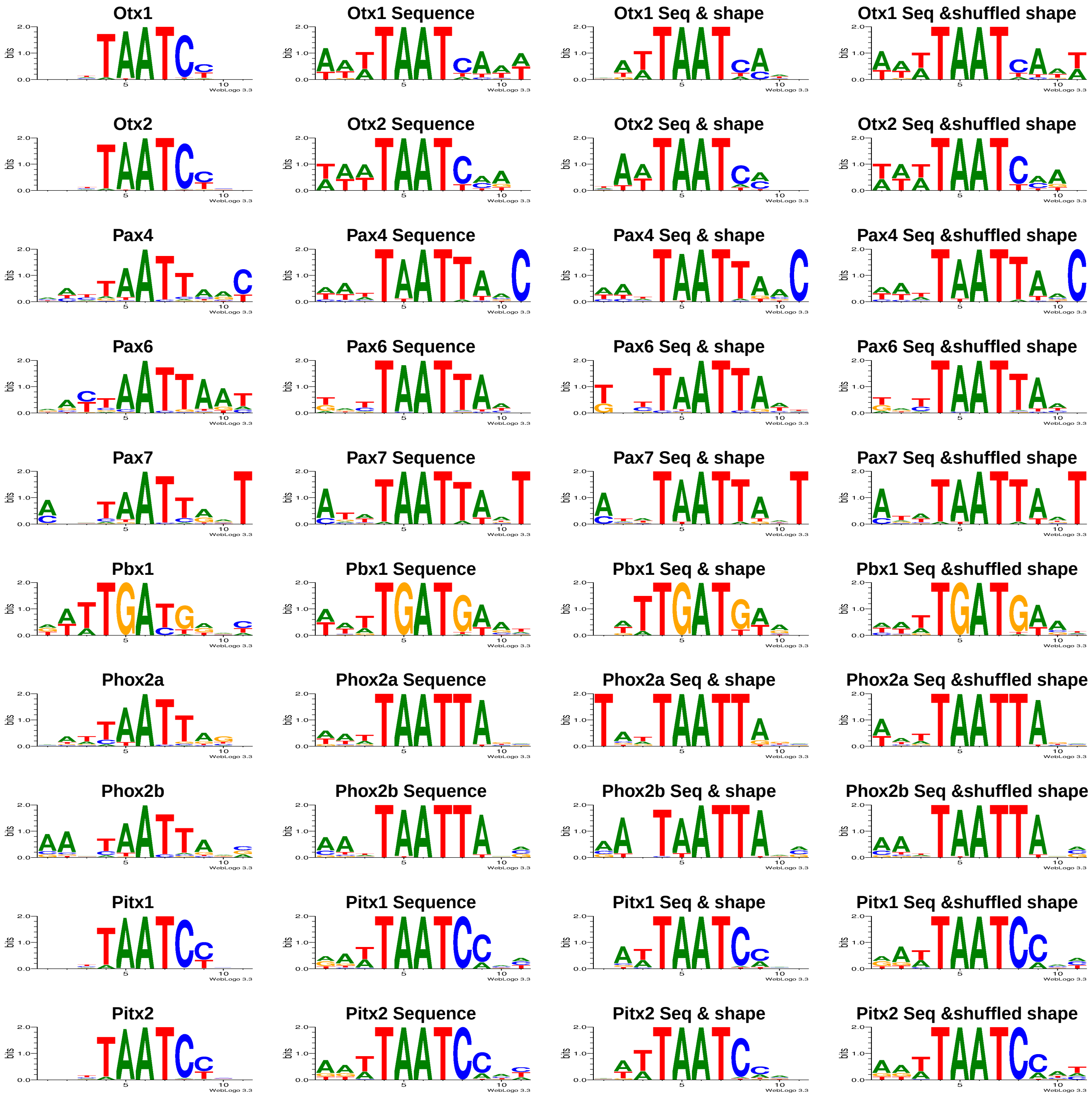
Otx1
Otx1 Sequence
Otx1 Seq & shape
Otx1 Seq &shuffled shape
Otx2
Otx2 Sequence
Otx2 Seq & shape
Otx2 Seq &shuffled shape
Pax4
Pax4 Sequence
Pax4 Seq & shape
Pax4 Seq &shuffled shape
Pax6
Pax6 Sequence
Pax6 Seq & shape
Pax6 Seq &shuffled shape
Pax7
Pax7 Sequence
Pax7 Seq & shape
Pax7 Seq &shuffled shape
Pbx1
Pbx1 Sequence
Pbx1 Seq & shape
Pbx1 Seq &shuffled shape
Phox2a
Phox2a Sequence
Phox2a Seq & shape
Phox2a Seq &shuffled shape
Phox2b
Phox2b Sequence
Phox2b Seq & shape
Phox2b Seq &shuffled shape
Pitx1
Pitx1 Sequence
Pitx1 Seq & shape
Pitx1 Seq &shuffled shape
Pitx2
Pitx2 Sequence
Pitx2 Seq & shape
Pitx2 Seq &shuffled shape

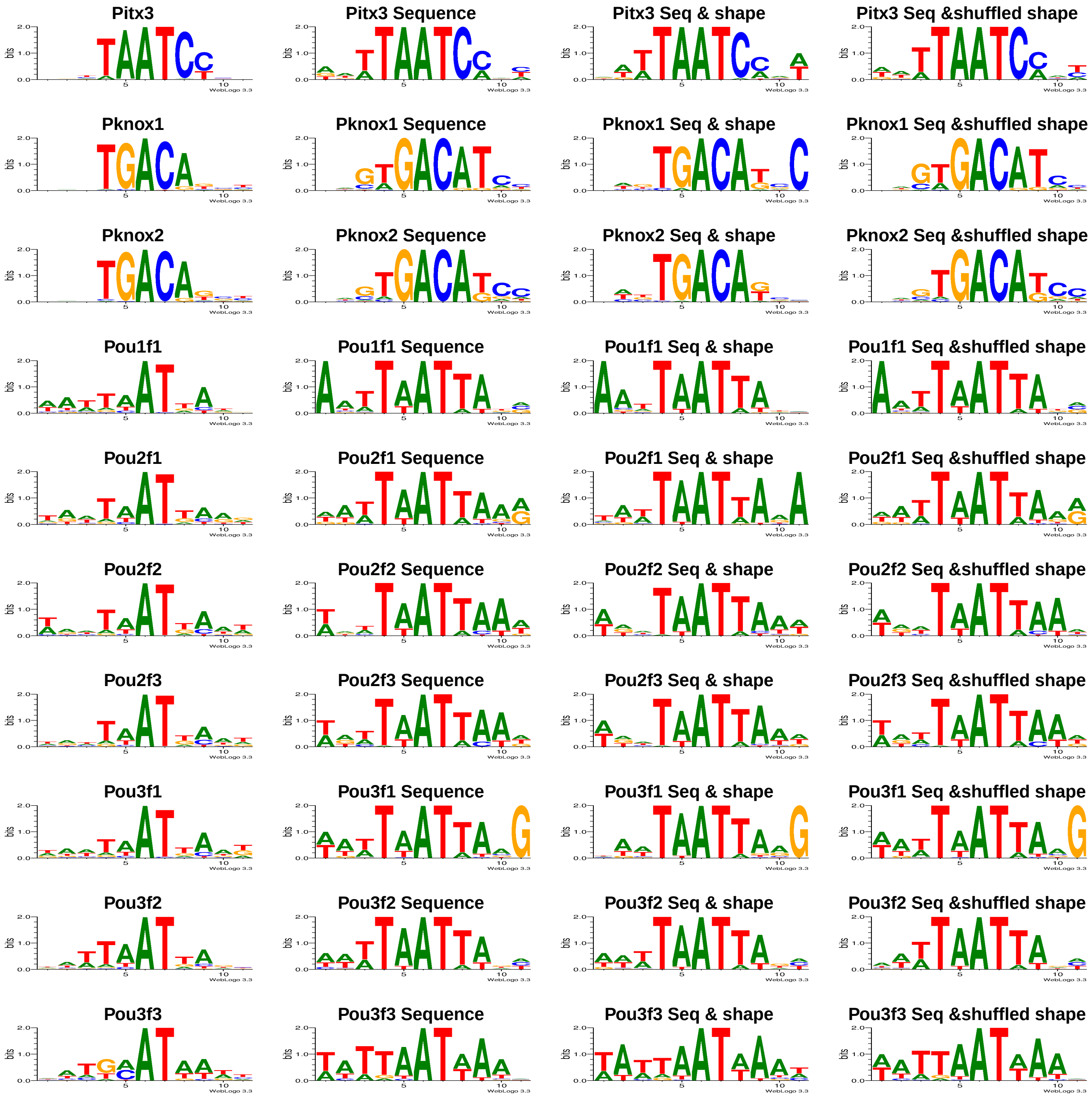
Pitx3
Pitx3 Sequence
Pitx3 Seq & shape
Pitx3 Seq &shuffled shape
Pknox1
Pknox1 Sequence
Pknox1 Seq & shape
Pknox1 Seq &shuffled shape
Pknox2
Pknox2 Sequence
Pknox2 Seq & shape
Pknox2 Seq &shuffled shape
Pou1f1
Pou1f1 Sequence
Pou1f1 Seq & shape
Pou1f1 Seq &shuffled shape
Pou2f1
Pou2f1 Sequence
Pou2f1 Seq & shape
Pou2f1 Seq &shuffled shape
Pou2f2
Pou2f2 Sequence
Pou2f2 Seq & shape
Pou2f2 Seq &shuffled shape
Pou2f3
Pou2f3 Sequence
Pou2f3 Seq & shape
Pou2f3 Seq &shuffled shape
Pou3f1
Pou3f1 Sequence
Pou3f1 Seq & shape
Pou3f1 Seq &shuffled shape
Pou3f2
Pou3f2 Sequence
Pou3f2 Seq & shape
Pou3f2 Seq &shuffled shape
Pou3f3
Pou3f3 Sequence
Pou3f3 Seq & shape
Pou3f3 Seq &shuffled shape

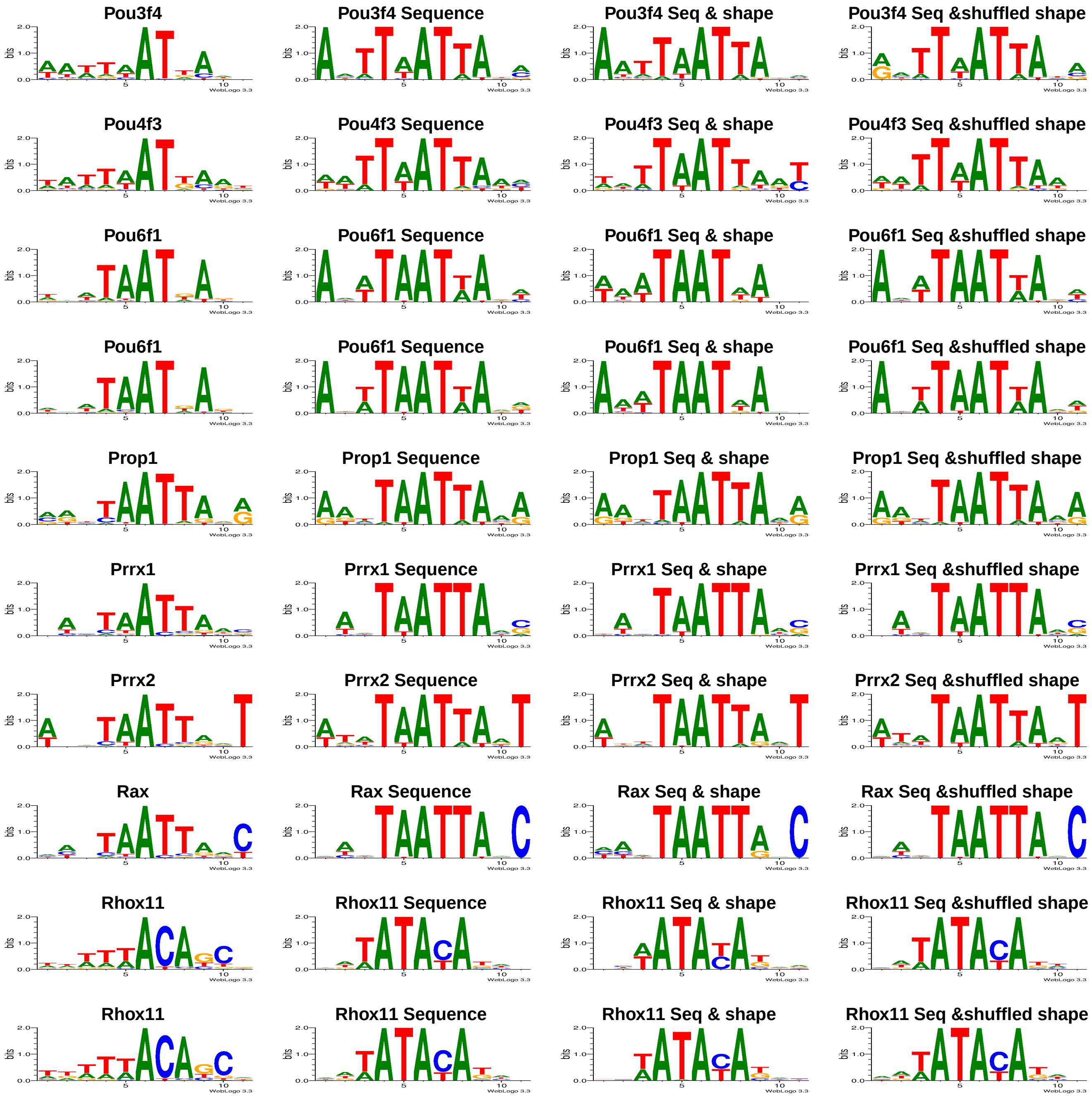
Pou3f4
Pou3f4 Sequence
Pou3f4 Seq & shape
Pou3f4 Seq &shuffled shape
Pou4f3
Pou4f3 Sequence
Pou4f3 Seq & shape
Pou4f3 Seq &shuffled shape
Pou6f1
Pou6f1 Sequence
Pou6f1 Seq & shape
Pou6f1 Seq &shuffled shape
Pou6f1
Pou6f1 Sequence
Pou6f1 Seq & shape
Pou6f1 Seq &shuffled shape
Prop1
Prop1 Sequence
Prop1 Seq & shape
Prop1 Seq &shuffled shape
Prrx1
Prrx1 Sequence
Prrx1 Seq & shape
Prrx1 Seq &shuffled shape
Prrx2
Prrx2 Sequence
Prrx2 Seq & shape
Prrx2 Seq &shuffled shape
Rax
Rax Sequence
Rax Seq & shape
Rax Seq &shuffled shape
Rhox11
Rhox11 Sequence
Rhox11 Seq & shape
Rhox11 Seq &shuffled shape
Rhox11
Rhox11 Sequence
Rhox11 Seq & shape
Rhox11 Seq &shuffled shape

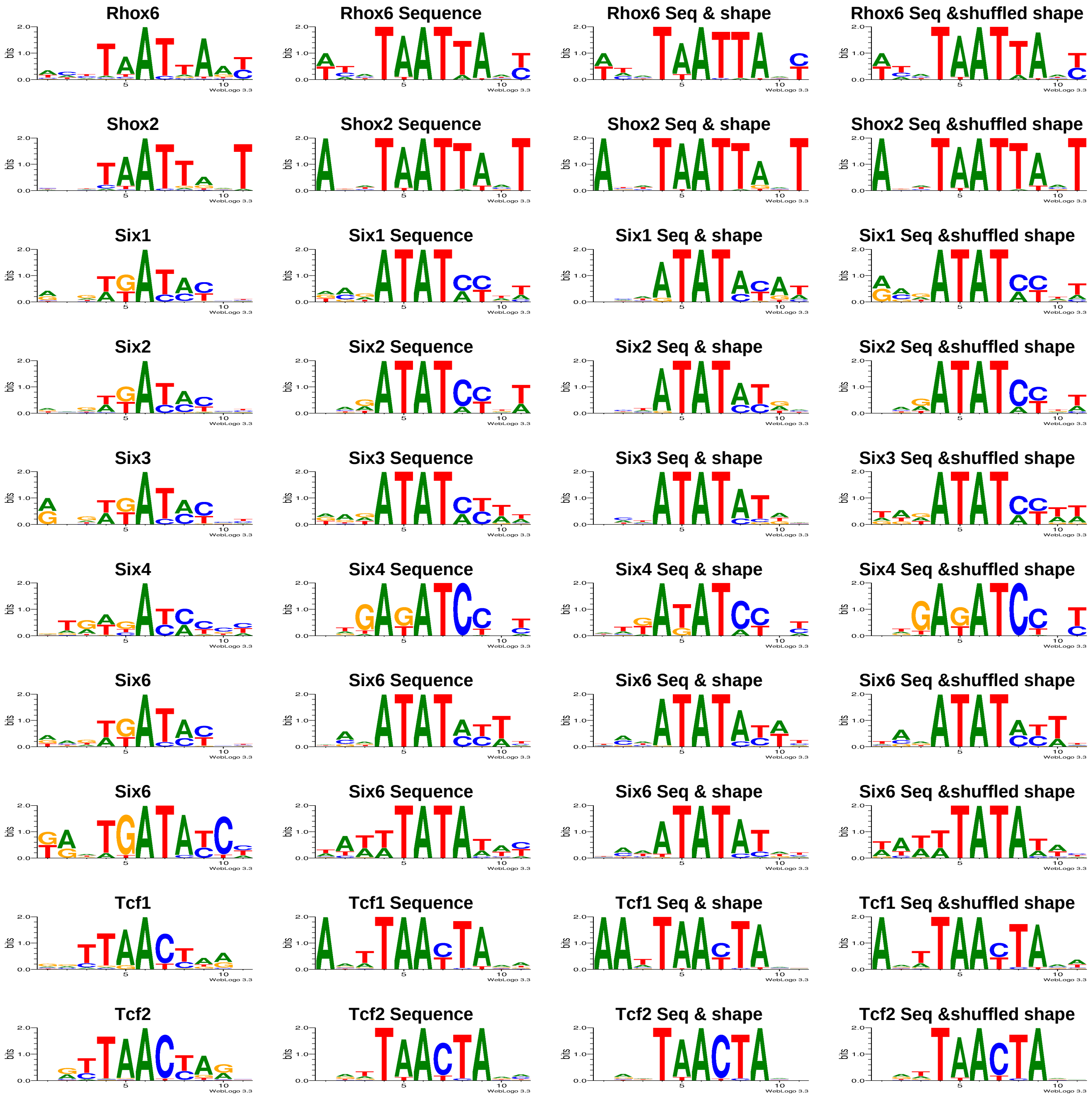
Rhox6
Rhox6 Sequence
Rhox6 Seq & shape
Rhox6 Seq &shuffled shape
Shox2
Shox2 Sequence
Shox2 Seq & shape
Shox2 Seq &shuffled shape
Six1
Six1 Sequence
Six1 Seq & shape
Six1 Seq &shuffled shape
Six2
Six2 Sequence
Six2 Seq & shape
Six2 Seq &shuffled shape
Six3
Six3 Sequence
Six3 Seq & shape
Six3 Seq &shuffled shape
Six4
Six4 Sequence
Six4 Seq & shape
Six4 Seq &shuffled shape
Six6
Six6 Sequence
Six6 Seq & shape
Six6 Seq &shuffled shape
Six6
Six6 Sequence
Six6 Seq & shape
Six6 Seq &shuffled shape
Tcf1
Tcf1 Sequence
Tcf1 Seq & shape
Tcf1 Seq &shuffled shape
Tcf2
Tcf2 Sequence
Tcf2 Seq & shape
Tcf2 Seq &shuffled shape

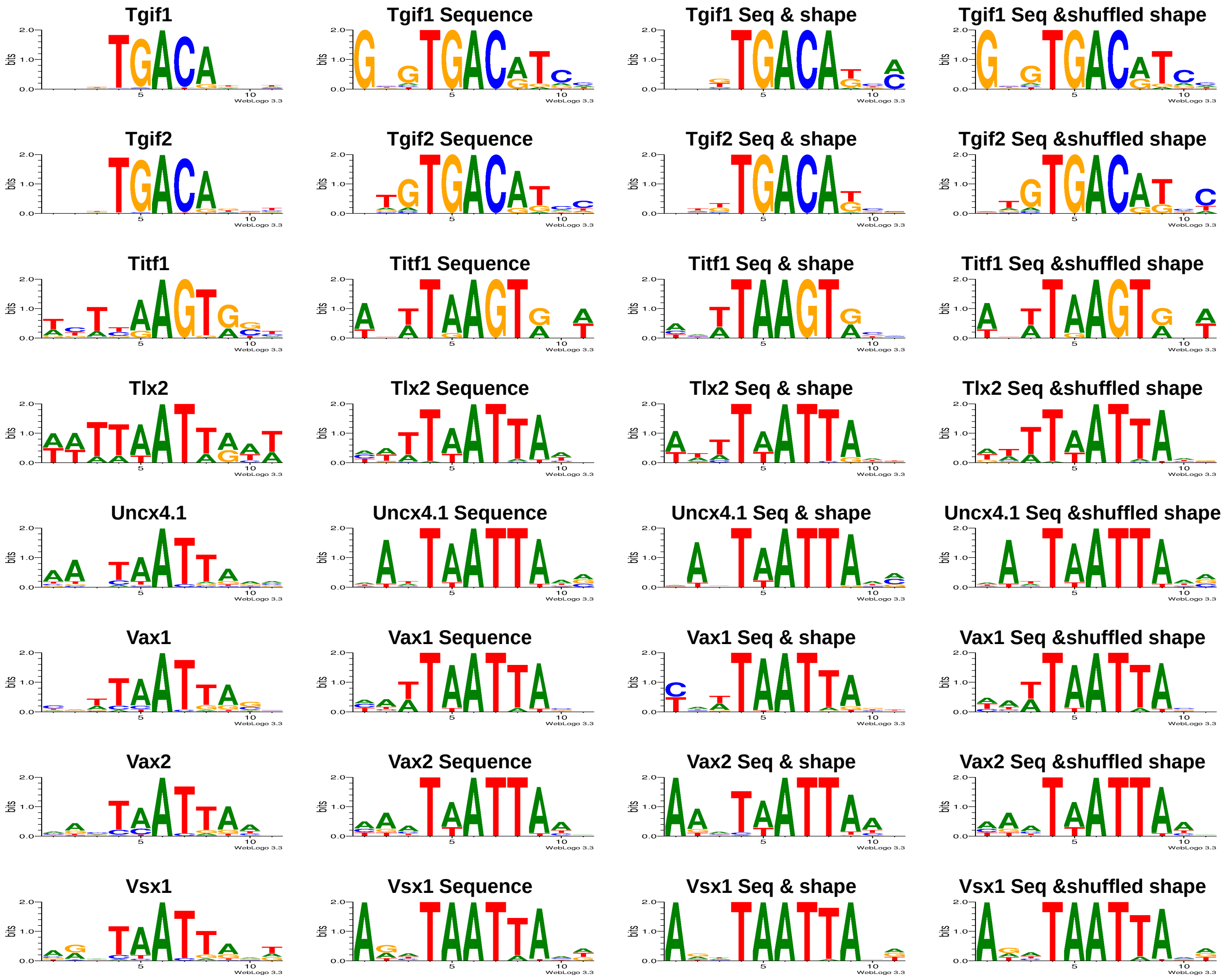
Tgif1
Tgif1 Sequence
Tgif1 Seq & shape
Tgif1 Seq &shuffled shape
Tgif2
Tgif2 Sequence
Tgif2 Seq & shape
Tgif2 Seq &shuffled shape
Titf1
Titf1 Sequence
Titf1 Seq & shape
Titf1 Seq &shuffled shape
Tlx2
Tlx2 Sequence
Tlx2 Seq & shape
Tlx2 Seq &shuffled shape
Uncx4.1
Uncx4.1 Sequence
Uncx4.1 Seq & shape
Uncx4.1 Seq &shuffled shape
Vax1
Vax1 Sequence
Vax1 Seq & shape
Vax1 Seq &shuffled shape
Vax2
Vax2 Sequence
Vax2 Seq & shape
Vax2 Seq &shuffled shape
Vsx1
Vsx1 Sequence
Vsx1 Seq & shape
Vsx1 Seq &shuffled shape
